# A conductive MXene hydrogel reprograms immunity and autophagy to restore neurovascular repair in infected wounds

**DOI:** 10.1093/rb/rbag108

**Published:** 2026-06-03

**Authors:** Yuanhui Xiao, Qingting Wu, Chunping Zeng, Yanyou Li, Kai Wang, Ziyu Peng, Yuner Luo, Zixi Wang, Lichun Huang, Shanshan Huang, Daolin Tang, Baoliang Zhang, Qiuyu Zhang, Jinbao Liu, Li Zhou

**Affiliations:** Guangdong Provincial Key Laboratory of Protein Modification and Disease, Key Laboratory of Biological Targeting Diagnosis, Therapy and Rehabilitation of Guangdong Higher Education Institutes, School of Basic Medical Sciences and The Fifth Affiliated Hospital, Guangzhou Medical University, Guangzhou 511436, P.R. China; Guangdong Provincial Key Laboratory of Protein Modification and Disease, Key Laboratory of Biological Targeting Diagnosis, Therapy and Rehabilitation of Guangdong Higher Education Institutes, School of Basic Medical Sciences and The Fifth Affiliated Hospital, Guangzhou Medical University, Guangzhou 511436, P.R. China; Guangdong Provincial Key Laboratory of Protein Modification and Disease, Key Laboratory of Biological Targeting Diagnosis, Therapy and Rehabilitation of Guangdong Higher Education Institutes, School of Basic Medical Sciences and The Fifth Affiliated Hospital, Guangzhou Medical University, Guangzhou 511436, P.R. China; Guangdong Provincial Key Laboratory of Protein Modification and Disease, Key Laboratory of Biological Targeting Diagnosis, Therapy and Rehabilitation of Guangdong Higher Education Institutes, School of Basic Medical Sciences and The Fifth Affiliated Hospital, Guangzhou Medical University, Guangzhou 511436, P.R. China; Guangdong Provincial Key Laboratory of Protein Modification and Disease, Key Laboratory of Biological Targeting Diagnosis, Therapy and Rehabilitation of Guangdong Higher Education Institutes, School of Basic Medical Sciences and The Fifth Affiliated Hospital, Guangzhou Medical University, Guangzhou 511436, P.R. China; Guangdong Provincial Key Laboratory of Protein Modification and Disease, Key Laboratory of Biological Targeting Diagnosis, Therapy and Rehabilitation of Guangdong Higher Education Institutes, School of Basic Medical Sciences and The Fifth Affiliated Hospital, Guangzhou Medical University, Guangzhou 511436, P.R. China; Nanshan School, Guangzhou Medical University, Guangzhou 511436, P.R. China; Guangdong Provincial Key Laboratory of Protein Modification and Disease, Key Laboratory of Biological Targeting Diagnosis, Therapy and Rehabilitation of Guangdong Higher Education Institutes, School of Basic Medical Sciences and The Fifth Affiliated Hospital, Guangzhou Medical University, Guangzhou 511436, P.R. China; Guangdong Provincial Key Laboratory of Protein Modification and Disease, Key Laboratory of Biological Targeting Diagnosis, Therapy and Rehabilitation of Guangdong Higher Education Institutes, School of Basic Medical Sciences and The Fifth Affiliated Hospital, Guangzhou Medical University, Guangzhou 511436, P.R. China; Guangdong Provincial Key Laboratory of Protein Modification and Disease, Key Laboratory of Biological Targeting Diagnosis, Therapy and Rehabilitation of Guangdong Higher Education Institutes, School of Basic Medical Sciences and The Fifth Affiliated Hospital, Guangzhou Medical University, Guangzhou 511436, P.R. China; Department of Surgery, UT Southwestern Medical Center, Dallas, TX 75390, USA; Key Laboratory of Special Functional and Smart Polymer Materials of Ministry of Industry and Information Technology, School of Chemistry and Chemical Engineering, Northwestern Polytechnical University, Xi’an 710129, P.R. China; Key Laboratory of Special Functional and Smart Polymer Materials of Ministry of Industry and Information Technology, School of Chemistry and Chemical Engineering, Northwestern Polytechnical University, Xi’an 710129, P.R. China; Affiliated Cancer Hospital & Institute of Guangzhou Medical University, Guangzhou Municipal and Guangdong Provincial Key Laboratory of Protein Modification and Disease, State Key Laboratory of Respiratory Disease, School of Basic Medical Sciences, Guangzhou Medical University, Guangzhou 511436, P.R. China; Guangdong Provincial Key Laboratory of Protein Modification and Disease, Key Laboratory of Biological Targeting Diagnosis, Therapy and Rehabilitation of Guangdong Higher Education Institutes, School of Basic Medical Sciences and The Fifth Affiliated Hospital, Guangzhou Medical University, Guangzhou 511436, P.R. China

**Keywords:** MXene microspheres hydrogel, immunomodulation, autophagy, cutaneous innervation, MRSA-infected wound healing

## Abstract

Multidrug-resistant bacteria-infected wounds are difficult to heal due to persistent infection, excessive inflammation, impaired angiogenesis and deficient cutaneous innervation. Here, we develop an antibacterial and conductive bioactive hydrogel based on flower-shaped MXene microspheres for treating methicillin-resistant *Staphylococcus aureus* (MRSA)-infected wounds. The hydrogel (PDM) is constructed by integrating **ε**-poly-L-lysine-functionalized MXene microspheres into a dynamically crosslinked oxidized pullulan network via pH-responsive Schiff-base chemistry, conferring injectability, self-healing, tissue adhesion and environmental responsiveness. PDM effectively eliminates MRSA biofilms, scavenges reactive oxygen species and attenuates inflammatory responses while promoting adaptive autophagy. These combined properties enable modulation of the wound microenvironment, enhance macrophage polarization toward a regenerative phenotype and support cell proliferation, endothelial cell migration and angiogenesis. In addition, the conductive hydrogel promotes Schwann cell maturation and neurotrophic factor expression, facilitating reconstruction of the neurogenic microenvironment. In a murine full-thickness MRSA-infected wound model, a single application of PDM significantly accelerates wound closure, enhances cutaneous innervation and reduces fibrosis. This work presents a multifunctional MXene-based hydrogel platform for antibiotic-free infected wound healing.

## Graphical Abstract

**Graphical Abstract:**
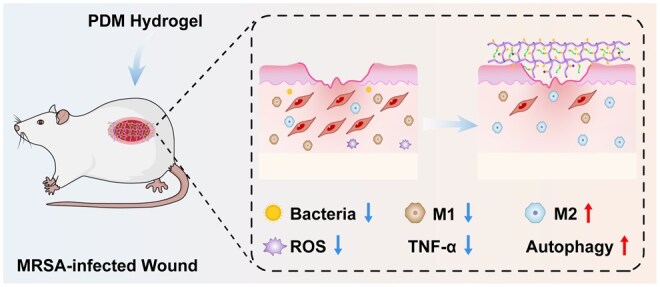

## Introduction

Multidrug-resistant bacteria-infected wounds remain a major global health challenge due to their high mortality rates and substantial socioeconomic burdens [1, 2]. Cutaneous wound healing is a highly coordinated physiological process involving dynamic interactions among multiple cell types and signaling pathways, classically progressing through four overlapping stages: hemostasis, inflammation, proliferation and tissue remodeling [3, 4]. In the presence of bacterial infection, however, this process is profoundly disrupted by a pathological wound microenvironment characterized by persistent microbial burden, excessive inflammation and accumulation of reactive oxygen species (ROS), resulting in delayed or failed healing [5, 6]. Moreover, inadequate cutaneous reinnervation and excessive fibrotic scar formation further compromise functional recovery, often leading to chronic non-healing wounds and long-term sensory dysfunction [7, 8]. Although antibiotics remain the standard treatment for infected wounds, their clinical efficacy is increasingly limited by adverse effects, poor bioavailability and the rapid emergence of drug resistance [9]. Methicillin-resistant *Staphylococcus aureus* (MRSA), one of the most prevalent and recalcitrant pathogens in wound infections, is difficult to eradicate and frequently causes prolonged immune dysregulation and unsatisfactory healing outcomes [10–12]. Therefore, the development of antibiotic-free therapeutic strategies for MRSA-infected wound healing is highly desirable.

Bioactive hydrogels have attracted considerable attention for infected wound management due to their favorable biocompatibility and capacity to integrate antibacterial and anti-inflammatory functions [5, 13–16]. Additionally, stimuli-responsive hydrogels that adapt to pathological cues such as pH, ROS or temperature offer opportunities to dynamically regulate the wound microenvironment during different stages of healing [17–19]. Beyond infection control and inflammation resolution, emerging evidence highlights autophagy as a critical regulatory process throughout wound repair. Autophagy is a lysosome-dependent intracellular recycling pathway that maintains cellular homeostasis and enhances stress tolerance under adverse conditions [20]. In the context of wound healing, autophagy exerts pleiotropic effects, including suppression of excessive inflammation, promotion of angiogenesis, facilitation of re-epithelialization and regulation of extracellular matrix remodeling [21–23]. In parallel, proper cutaneous innervation is increasingly recognized as an essential determinant of high-quality wound repair, as impaired nerve regeneration can result in incomplete healing, chronic pain, pruritus and sensory loss [24, 25]. However, most existing bioactive hydrogels primarily focus on antibacterial activity and inflammation control, while largely neglecting coordinated regulation of autophagy and neural regeneration. To date, few strategies have successfully integrated immunomodulation, autophagy regulation and cutaneous innervation to promote complete healing of MRSA-infected wounds.

MXene, a type of 2D transition metal carbides, nitrides and carbonitrides with a general formula of M_n+1_X_n_Tₓ (*n* = 1–3), has emerged as a promising biomaterial due to its unique physicochemical properties [26, 27]. MXene nanosheets exhibit excellent mechanical properties, biocompatibility, biodegradability, antibacterial and antioxidant properties [26, 28–30]. Additionally, Ti_3_C_2_ MXene can improve the autophagy level of human umbilical vein endothelial cells (HUVECs) or HTR-8/SV neo cells through PI3K/AKT/mTOR pathway [31, 32], while their high electrical conductivity enables them to mimic endogenous bioelectric cues that regulate cell migration, angiogenesis and nerve regeneration [33, 34]. These features suggest strong potential for MXene-based materials in comprehensive wound repair. Nevertheless, pristine Ti_3_C_2_Tₓ nanosheets are prone to interlayer restacking and oxidative degradation, and their limited tissue adhesion hampers long-term retention at wound sites, thereby restricting therapeutic efficacy [35]. To overcome these limitations, assembling MXene nanosheets into flower-shaped microspheres is an effective strategy. Functionalizing them with ε-poly-L-lysine (EPL) also enhances their structural stability, antibacterial performance and tissue compatibility [36]. The flower-shaped EPL-modified MXene microspheres (MPL) present several advantages over traditional MXene nanosheets in wound-healing. Due to their 3D architecture, MPL are less susceptible to aggregation and restacking, which aids in preserving the accessible surface area and maintaining functional performance. In comparison with MXene nanosheets, the microspherical morphology of MPL may also alleviate mechanical irritation to cells and tissues, thereby enhancing biocompatibility and biosafety. Additionally, MPL generally demonstrates enhanced stability in the moist wound microenvironment, presumably owing to the partial self-protective effect of their inner layered structure, which contributes to improved dispersion stability, slower oxidation and prolonged functional durability. Their 3D particulate configuration is also beneficial for incorporation into wound-dressing matrix, as it can augment internal porosity, facilitate moisture vapor exchange and exudate management, and provide a more favorable microenvironment for cell migration, adhesion and proliferation. Furthermore, the larger size and more integrated structure of MPL enable easier immobilization within hydrogel matrix, thereby reducing material loss, restricting undesired tissue penetration and enhancing localized therapeutic action. Additionally, incorporating these EPL-modified MXene microspheres (MPL) into hydrogel matrix may also successfully achieve functional integrations to promote infected-wound healing. However, the roles of MPL-based hydrogels in coordinating immunomodulation, autophagy regulation, cutaneous innervation and complete MRSA-infected wound healing remain largely unexplored.

In this study, we developed an antibacterial and conductive flower-shaped MXene microsphere-based hydrogel (PDM) with pH/redox responsiveness, self-healing capability, tissue adhesion and hemostatic properties for MRSA-infected wound repair. PDM was fabricated via dynamic Schiff-base crosslinking among oxidized pullulan (PCHO), MPL and 3,3′-dithiobis(propionohydrazide) (DTPH), which could release active components in response to changes in the microenvironment, avoiding toxicity caused by excessive local concentration and achieving continuous regulation of the wound repair process. In this network, MPL was endowed with antibacterial, antibiofilm, antioxidant, anti-inflammatory and biological activities. DTPH was utilized to attain ROS responsiveness owing to the disulfide bonds in its structure. PCHO, as the principal component of PDM hydrogel, provided the hydrogel matrix and antibacterial effects, which formed the gel network through Schiff base reaction between its aldehyde group and the amino groups of MPL and DTPH. *In vitro* studies demonstrated that PDM effectively eliminated MRSA biofilms, scavenged ROS, attenuated inflammatory responses and enhanced autophagy in endothelial cells, thereby promoting cell migration and angiogenic tubule formation. Furthermore, PDM upregulated the expression of wound-healing-related genes, including vascular endothelial growth factor (VEGF), angiogenin (*ANG*), smooth muscle α-actin (α-actin) and collagen type III (Col III), while stimulating Schwann cell morphogenetic maturation and neurotrophic secretory function. *In vivo*, PDM suppressed MRSA infection, promoted M2 macrophage polarization, enhanced autophagy-driven angiogenesis and re-epithelialization, reduced fibrosis and markedly improved cutaneous innervation. Collectively, these findings support the hypothesis that PDM promotes MRSA-infected wound healing through synergistic regulation of immunity, autophagy and neural regeneration.

## Materials and methods

### Preparation and characterization of PDM hydrogel

PCHO was prepared as described previously [37]. Flower-shaped Ti_3_C_2_T_x_ MXene@EPL microspheres (MPL) were fabricated using a similar approach to reported previously (presented in the Supplementary data) [38, 39]. PDM was obtained by Schiff base reaction between MPL, DTPH and PCHO. Briefly, the dispersed solution 5 wt% MPL solution, 2.5 wt% DTPH solution and 10 wt% PCHO solution were prepared. Then, MPL solution (5 μL) and DTPH (5 μL) solution were added to PCHO solution (90 μL) at room temperature. After mixing, PDM hydrogel was obtained. PD hydrogel (without MPL) was used as control. The formulations of PDM hydrogel and controls were showed in Supplementary Table S1. The characterizations of PDM and controls were performed using Fourier transform infrared spectroscopy (FTIR) and scanning electron microscope (SEM, Verios G4, FEI), respectively. The structure and elemental mapping of MPL were examined by SEM equipped with an energy-dispersive spectrometer (EDS).

### Evaluation of multifunctional properties

The pH/redox-responsiveness, self-healing capability, stability, conductivity, tissue adhesion, swelling ratio, degradation behavior and hemostatic properties of PDM were evaluated as outlined previously [40, 41]. The rheological characteristics of PDM, including the storage modulus (G′), loss modulus (G′′) and oscillation strain and viscosity were tested using a TA rheometer (DHR-2). Briefly, the G′ and G″ of PDM and controls were recorded at 4°C, 25°C and 37°C. The oscillation strain, ranging from 1% to 1000% (three cycles), was performed at 37°C and 1 Hz frequency. Meanwhile, the viscosity of PDM and its controls was measured at various shear rates (0–20 s^−1^). PCHO and PD were employed as controls for assessing the contributions of the primary component (PCHO) and hydrogel matrix (PD) to the multifunctional properties of PDM, respectively. The detailed methods are provided in the Supplementary data.

### Antibacterial, antioxidant, anti-inflammatory effects and cytotoxicity *in vitro*

The antibacterial efficacy of PDM hydrogel was assessed by evaluating the viability of *Escherichia coli* (*E. coli*), *Staphylococcus aureus* (*S. aureus*) and MRSA, following incubation on the hydrogel’s surface as previously reported [41]. Additionally, the antibiofilm effect of PDM against MRSA biofilms was evaluated using the LIVE/DEAD® BacLight™ Bacterial Viability Kit as previously described. The stained biofilms were then imaged using a confocal fluorescence microscope (Zeiss LSM800). Dead bacteria exhibited red fluorescence, whereas viable ones appeared green [42].

The antioxidant capacity of PDM was assessed through •OH and DPPH scavenging tests as previously described [43, 44]. In brief, 100 μL PDM hydrogel or 5 μL MPL solution (50 mg/mL) was combined and incubated with a solution (100 μL) containing methylene blue (MB, 10 μg/mL), H_2_O_2_ (1 M) and FeCl_2_⋅4H_2_O (0.2 mg/mL). The MB concentration for each group was determined by measuring the absorption of the solution at 666 nm. To evaluate the DPPH free radical scavenging ability of PDM, 100 μL of diluted PDM solutions at varying concentrations (0.4, 0.8 and 1.6 mg/mL) were co-incubated with a DPPH solution (100 μL, with a final concentration of 50 µM) in the dark for 15 min following stirring. A vitamin C solution (2 mg/mL) treated DPPH solution served as a positive control. Subsequently, the absorbance of the mixture was measured at 517 nm utilizing a UV-vis spectrophotometer, and the DPPH degradation ratio was calculated based on these absorbance readings [44]. The ability of PDM to eradicate intracellular ROS was analyzed in L929 cells. The cells were cultured in a 24-well plate. After adhering, H_2_O_2_ (final concentration of 200 μM) and the diluted PDM solution (final concentration of 20 μg/mL) or controls were added and then co-incubated for 24 h. Subsequently, the cells were stained with DCFH-DA at a concentration of 10 μM, and images were taken by an inverted fluorescence microscope (IX53, Olympus).

The anti-inflammatory activity of PDM was evaluated in RAW 264.7 cells. Briefly, RAW 264.7 cells were plated in a 6-well dish and exposed to 1 μg/mL LPS for 24 h following adherence. Following this, the culture medium was substituted with a fresh medium including the diluted PDM solution (final concentration of 20 μg/mL), and the cells were incubated for an additional 24 h. Subsequently, the cells were collected and co-incubated with CD86-PE-Cy7 (BD Pharmingen PE-Cy7 Rat Anti-Mouse CD86, GL1, 560582, labeled as M1) and CD206-Alexa Fluor 647 antibodies (BD Pharmingen Alexa Fluor 647 Rat Anti-Mouse CD206, MR5D3, 565250, labeled as M2) at 4°C for 30 min. Flow cytometry was employed to investigate the macrophage polarization. The NC, LPS and PD groups were, respectively, designated as the negative control (NC) group, the model group and the hydrogel matrix group. Concurrently, the mRNA expression levels of TNF-α and IL-10 in RAW 264.7 cells were determined using *qRT-PCR*.

HUVECs, L929, NIH 3T3 and RSC-96 cells were co-cultured with diluted solutions of PCHO, MPL, DTPH, PD and PDM (ranging from 20 to 80 μg/mL) to evaluate their cytotoxicity. After co-incubating for 24 h, cell viability was determined using a CCK8 kit following the manufacturer’s protocol.

### Evaluation of autophagy, cell proliferation and migration *in vitro*

To investigate cell autophagy, HUVECs were plated in 6-well plates with 15 mm coverslips (4.5 × 10^4^ cells/well). After adherence, the culture medium was replaced with fresh medium containing the diluted PDM solution or controls (final concentration 20 μg/mL) and co-incubated for 24 h. Immunofluorescence staining was used to analyze the autophagy levels in HUVECs following various treatments. Meanwhile, the expressions of LC3 and p62, which are associated with autophagy, were measured using western blotting (WB).

To assess cell proliferation, L929 cells were co-incubated with a diluted PDM solution (final concentration of 20 μg/mL) or control solutions for 1, 3 and 5 days, with the culture medium being changed every two days. At the designated times, the morphology and viability of L929 cells were detected by LIVE/DEAD kit and Alamar Blue® kit, respectively. The stained cells were subsequently captured with a fluorescence microscope (IX53, Olympus).

The impact of PDM on cell migration was evaluated by cell scratch assay as previously reported [40]. Briefly, HUVECs were seeded in a mold within a 6-well plate at a concentration of 1.5 × 10^5^ cells/mL and cultured for 12 h. Then, the mold was removed and the cells were rinsed with PBS before adding the diluted PDM solution (final concentration of 20 μg/mL) or controls. After treatment at 0, 12 and 24 h, the scratch areas of all groups were imaged, and the migration rate was quantitatively assessed utilizing ImageJ software.

Each component of the PDM and PD was used as controls for assessing the influence of them on autophagy, cell proliferation and migration.

### Angiogenesis, gene expressions and Schwann cell maturation *in vitro*

The effect of PDM on vascularization was examined through an endothelial tube formation assay. In brief, 50 μL pre-cooled Matrigel matrix was uniformly distributed across 24-well plate and then kept at 37°C for 30 min. Following this, a combination of HUVECs and a diluted PDM solution (final concentration of 20 μg/mL) or control samples were individually cultured on the Matrigel matrix. After co-culture for 8 h, the formation of tubes within each group was investigated using an inverted microscope. Subsequently, the ‘Angiogenesis Analyzer’ tool available in ImageJ software was conducted to statistical analysis. In addition, the relative gene expressions of VEGF, *ANG*, α-actin and Col III were quantified by *qRT-PCR* in HUVECs and NIH 3T3 cells after treatment with a diluted PDM solution (final concentration of 20 μg/mL) or controls for 3 days, respectively.

To evaluate the impact of PDM hydrogel on the maturation and secretion ability of Schwann cell, RSC-96 cells were grown in a 24-well plate (20 000 cells/well) or on cell slides for 18 h, respectively. Subsequently, the culture medium was changed with fresh medium including a diluted PDM solution (final concentration 20 μg/mL) or controls. After co-incubation for 3 days, immunofluorescence staining of S100 was performed on the cells on the cell slide to assess the morphological maturation of Schwann cell. The stained cells were photographed by Laser Scanning Confocal Microscope (ZEISS 900). Meanwhile, the expression levels of S100*, PMP22*, NCAM and key neurotrophic factor genes, containing *BDNF*, NGF and *CNTF* were examined using *qRT-PCR* for investigating the genetic modulation of PDM hydrogel on RSC-96 cells as described previously [45]. Similarly, each component of the PDM and PD were employed as controls for evaluating the impact of them on vascularization and Schwann cell maturation.

Additionally, to investigate the role of enhanced autophagy in the proangiogenic or proneurogenic effects of PDM, the *in vitro* angiogenesis and Schwann cell maturation of PDM under autophagy inhibition were assessed. Specifically, HUVECs were cultured on the Matrigel matrix with a diluted PDM solution (with a final concentration of 20 μg/mL) and a Bafilomycin A1 solution (BafA1, an autophagy inhibitor, final concentration of 100 nM). The tube formation in the NC and PDM+BafA1 groups was examined as previously described. For the Schwann cell maturation assay, RSC-96 cells were treated with a diluted PDM solution (final concentration 20 μg/mL) and a BafA1 solution (final concentration of 100 nM). Subsequently, immunofluorescence staining of S100 was carried out as described above.

### *In vivo* anti-infection and MRSA-infected wound healing

The MRSA-infected full-thickness cutaneous wound model was created utilizing Kunming mice (female, 28–32 g, *n* = 6, Vital Rive, Guangdong, China). The procedures of animal experiments were approved by the laboratory animal ethics committee of Guangzhou Medical University (No. GY2024-887). The MRSA-infected wound model was established in a similar method to previously described [41]. Briefly, following anesthesia and shaving, two round full-thickness cutaneous wounds (diameter 7 mm) were formed on the back of each mouse. Then, 10 μL of MRSA solution (10^6^ CFU mL^−1^) was introduced to each wound site. Two hours later, 100 μL of PDM hydrogel was applied to the wound area, which was subsequently covered with a hollow Tegaderm Film (3M) to prevent wound contraction during the healing phase. Control groups, consisting of Blank (no treatment, NC), 3M (without hollow, positive control) and PD group (hydrogel matrix), were also created for comparative analysis. On Days 0, 3, 7 and 14, the wounds were documented using a digital camera, and their sizes were measured with ImageJ software. Tissue samples were excised and split into two portions on Days 3, 7 and 14. One portion of the tissues was homogenized and diluted with PBS to an appropriate ratio (1:100), then plated on LB agar to assess the anti-infective effect at the wound site. The remaining portion was subjected to Hematoxylin and Eosin (H&E) staining, Masson’s trichrome staining and immunofluorescence staining (including F4/80, Ly6G, TNF-α, IL-10, iNOS, CD206, LC3B, Ki67, CD31, α-SMA, Col I and Cytokeratin) to analyze infection, inflammatory infiltrate, macrophage polarization, autophagy, proliferation, angiogenesis, fibrosis, collagen deposition and epithelialization during the wound healing process. Concurrently, to evaluate the possible mechanism and innervation in the wound area, immunostaining of alpha 7-nAChR (marker: α-Bungarotoxin, α-BTX) and nerves (marker: βIII-tubulin) was performed. All sections were imaged and analyzed with panoramic digital slide scanners (3DHISTEC, Pannoramic MIDI) and ImageJ software. Additionally, the transcriptomic analysis of regenerated MRSA-infected wound tissues on Day 7 was conducted by Beijing SeekGene BioSciences Co., Ltd. using Illumina Novaseq 6000 for sequencing. The gene ontology terms, signaling pathway terms and differential gene expression were analyzed.

### Statistical analysis

Data were expressed as mean±SD values from at least *n* = 3 experiments. Differences between groups were analyzed for statistical significance with unpaired two-tailed Student’s *t*-tests or one-way analysis of variance (one-way ANOVA) with *post hoc* Tukey’s multiple comparison test, respectively. All statistical analyses were conducted with GraphPad Prism 10.1.2 Software. The criterion for statistical significance was defined at *P* < 0.05, represented as *P < 0.05, **P < 0.01, ***P < 0.001 and ‘ns’ signifies nonsignificant differences. *In vivo* experiments, n represents the number of wounds and the analysis was performed by wound.

## Results and discussion

### Preparation and characterization of PDM hydrogel

**Figure 1:**
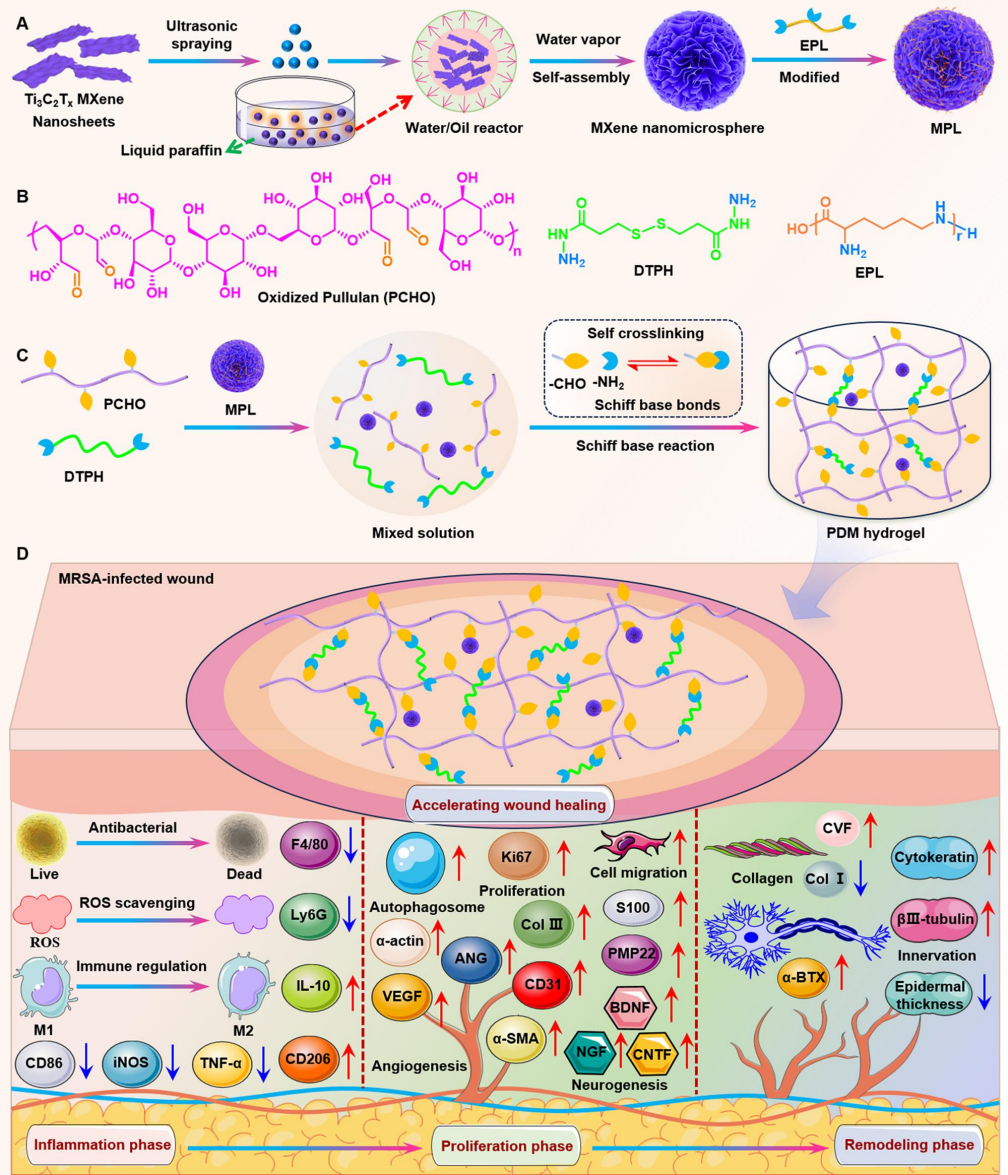
Schematic illustration showing the fabrication and application of PDM hydrogel in MRSA-infected wound healing. (**A**) The preparation process of EPL-modified flower-shaped MXene microspheres (MPL). (**B**) Structure of PCHO, DTPH and EPL. (**C**) Schematic illustration for the formation of PDM hydrogel by Schiff base reaction. (**D**) The application of PDM hydrogel in MRSA-infected wound healing.

The flower-shaped Ti_3_C_2_T_x_ MXene microspheres were fabricated via an ultrasonic spray-assisted assembly process. The resulting microspheres were subsequently functionalized with EPL through electrostatic adsorption to obtain MPL, as detailed in the Supplementary data. PCHO was synthesized following a previously reported protocol [37]. PDM hydrogel was then constructed via a Schiff-base reaction between the aldehyde groups of PCHO and the amino groups of MPL and DTPH, respectively (Figure 1). A hydrogel matrix prepared in the absence of MPL was used as a control and denoted as PD.

**Figure 2:**
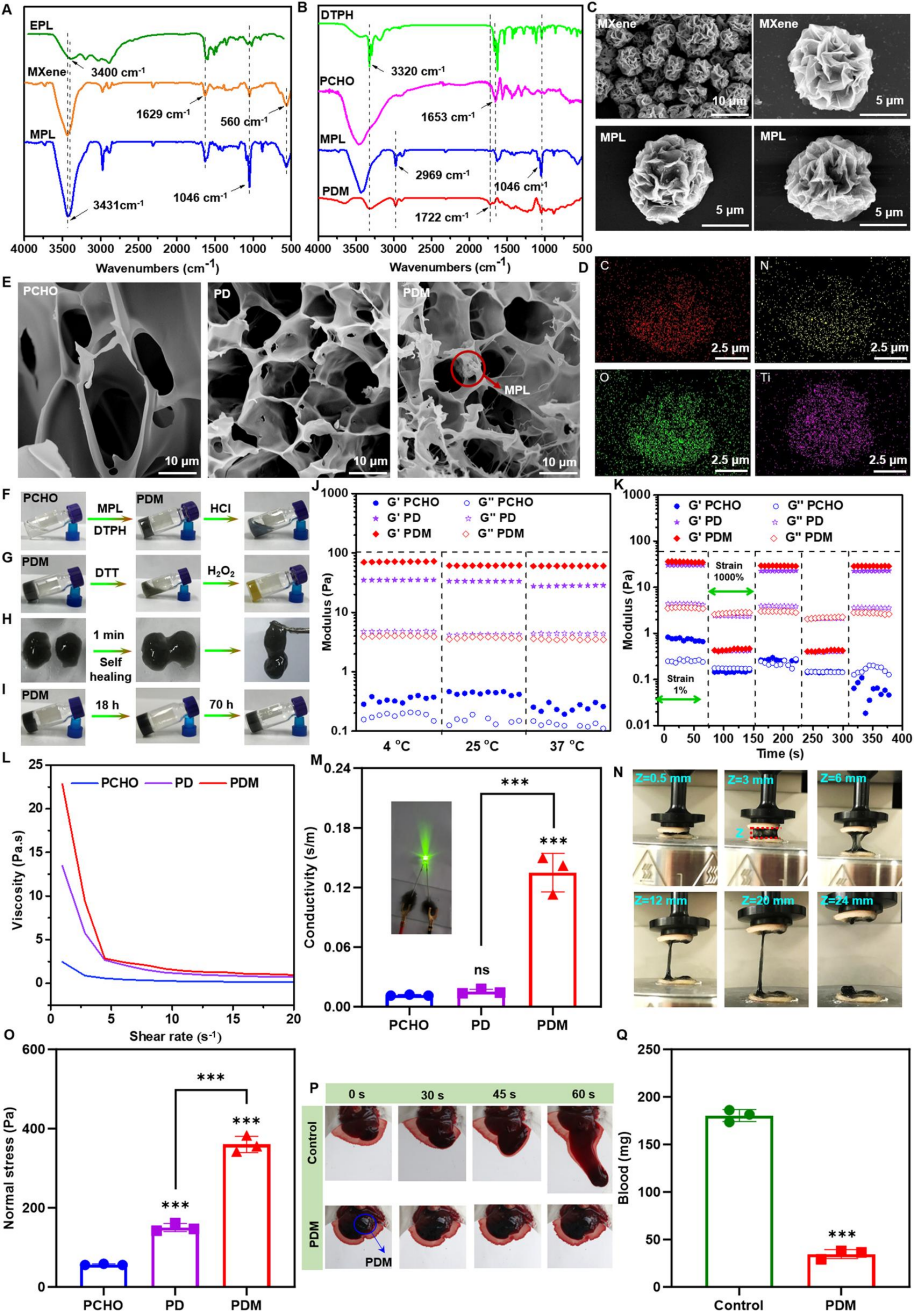
Structure characterization and multifunctional properties of PDM and controls. (**A, B**) FTIR of EPL, MXene, MPL, DTPH, PCHO and PDM. (**C**) SEM images of flower-shaped Ti_3_C_2_T_x_ MXene microspheres and MPL. (**D**) Elemental mapping of MPL including C, N, O and Ti. (**E**) SEM images of the freeze-dried samples of PCHO, PD and PDM. (**F**) Gel formation and pH-responsive test images of PDM. (**G**) Images of redox-responsive test of PDM. (**H**) Pictures of the self-healing of PDM. (**I**) Pictures of the PDM hydrogel placed at room temperature for different times. (**J**) Rheological analysis of PDM hydrogel and controls at 4°C, 25°C and 37°C. (**K**) Rheological assay of PCHO, PD and PDM under alternating high (1000%) and low shear strain (1%). (**L**) Viscosity of PDM and controls at different shear rate. (**M**) The conductivity property of PDM and controls. Inset: image showing that PDM hydrogel served as a conductor to connect the circuit and LED light. (**N**) Images of PDM adhering to fresh porcine skin. (**O**) Normal stress of PDM and controls on fresh porcine skin. (**P, Q**) Images of hemorrhaging site at different times and total blood loss from the damaged livers after treatment with PDM or control for 60 s (***P < 0.001 and ns means not significant differences. *n* = 3).

The structures of EPL, flower-shaped Ti_3_C_2_T_x_ MXene microspheres, MPL, DTPH, PCHO and PDM were characterized by FTIR. As shown in Figure 2A, EPL exhibited a characteristic absorption band at 3400 cm^−1^ corresponding to amino group stretching vibrations. Pristine MXene microspheres displayed distinct absorption bands at 3431 cm^−1^ (–OH), 1629 cm^−1^ (Ti–O), 1046 cm^−1^ (C–O) and 560 cm^−1^ (C–F), all of which were retained in the MPL spectrum, confirming successful EPL functionalization. In Figure 2B, DTPH showed a characteristic N–H stretching vibration at 3320 cm^−1^, while PCHO exhibited a pronounced aldehyde C = O stretching band at 1653 cm^−1^. The absorption bands at 2969 and 1046 cm^−1^ were attributed to the C–H stretching vibration and C–O bond of MPL, respectively. In the PDM spectrum, the disappearance of aldehyde absorption band (1653 cm^−1^) and emergence of stretching vibration of C = N (1722 cm^−1^) confirmed the formation of the Schiff-base-crosslinked hydrogel network.

The microstructures of Ti_3_C_2_Tₓ MXene microspheres and MPL were further examined by SEM. As shown in Figure 2C, Ti_3_C_2_T_x_ MXene assembled into well-defined flower-shaped microspheres, and this morphology was preserved after EPL modification. Element mapping analysis further verified the successful preparation of MPL microspheres (Figure 2D). Additionally, SEM images revealed that both PD and PDM exhibited interconnected porous structures. Notably, MPL microspheres were clearly embedded within the PDM hydrogel matrix, confirming their successful incorporation (Figure 2E).

### Evaluation of multifunctional features

The PDM hydrogel was constructed by dynamically crosslinking PCHO, DTPH and MPL through the Schiff-base reaction (Figure 2F). Its multifunctional features-including pH/redox responsiveness, self-healing capability, stability, rheological characteristics, viscosity, conductivity, tissue adhesion and hemostatic ability-were summarized in Figure 2F–Q. Upon exposure to HCl, PDM underwent a reversible gel-to-sol transition due to disruption of Schiff-base linkages, confirming its pH responsiveness (Figure 2F). Similarly, a gel-sol-gel transition was achieved through sequential treatment with dithiothreitol (DTT) and hydrogen peroxide (H_2_O_2_), reflecting redox responsiveness mediated by disulfide bonds within the network (Figure 2G). PDM also exhibited rapid self-healing behavior, as physically separated hydrogel fragments readily rejoined within 1 min upon contact (Figure 2H). The hydrogel maintained structural integrity for at least 70 h at room temperature, demonstrating good stability (Figure 2I). Rheological analysis revealed that the storage modulus (G′) of both PDM and PD exceeded the loss modulus (G′′) over a temperature range of 4–37°C, confirming successful hydrogel formation (Figure 2J). Notably, PDM displayed higher G′ and G′′ values than PD, indicating reinforcement of the hydrogel network by MPL incorporation. Under alternating high (1000%) and low (1%) shear strains, both PDM and PD showed pronounced shear-thinning behavior and rapid recovery of G′ and G′′ over multiple cycles, indicative of robust self-healing capability (Figure 2K). In addition, the viscosities of PDM and PD were consistently higher than those of PCHO across a wide range of shear rates, confirming covalent network formation rather than physical mixing (Figure 2L).

Benefiting from the intrinsic conductivity of MPL, PDM exhibited significantly enhanced electrical conductivity compared with PD, enabling it to function as a conductive bridge in an electrical circuit to illuminate a light-emitting diode (Figure 2M). Moreover, PDM demonstrated strong adhesion to fresh porcine skin, with substantially higher normal stress values than control hydrogels, highlighting its suitability for stable wound-site retention (Figure 2N and O). The swelling ratio of PDM hydrogel was maintained at 36.6 after being immersed in PBS for 120 min, which was conducive to the absorption of wound exudate (Supplementary Figure S1A). The degradation behavior of PDM was of great significance for its biological applications. As depicted in Supplementary Figure S1B, the degradation rate of PDM hydrogel increased in PBS (pH = 7.4) with the passage of time at room temperature. PDM was nearly 100% degraded after being fully immersed in PBS for 11 h. The hemostatic performance of PDM was further evaluated using a mouse liver hemorrhage model. Compared with untreated controls, which exhibited severe bleeding (180.4 ± 6.32 mg after 60 s), PDM treatment rapidly arrested hemorrhage, reducing blood loss to 34.6 ± 4.74 mg (Figure 2P and Q).

Collectively, these results demonstrated that PDM integrates dynamic responsiveness, mechanical robustness, electrical conductivity, strong tissue adhesion and rapid hemostatic activity, providing a multifunctional platform capable of establishing a favorable microenvironment for infected wound healing.

### Antibacterial, antioxidant, anti-inflammatory activities and cytotoxicity *in vitro*

**Figure 3:**
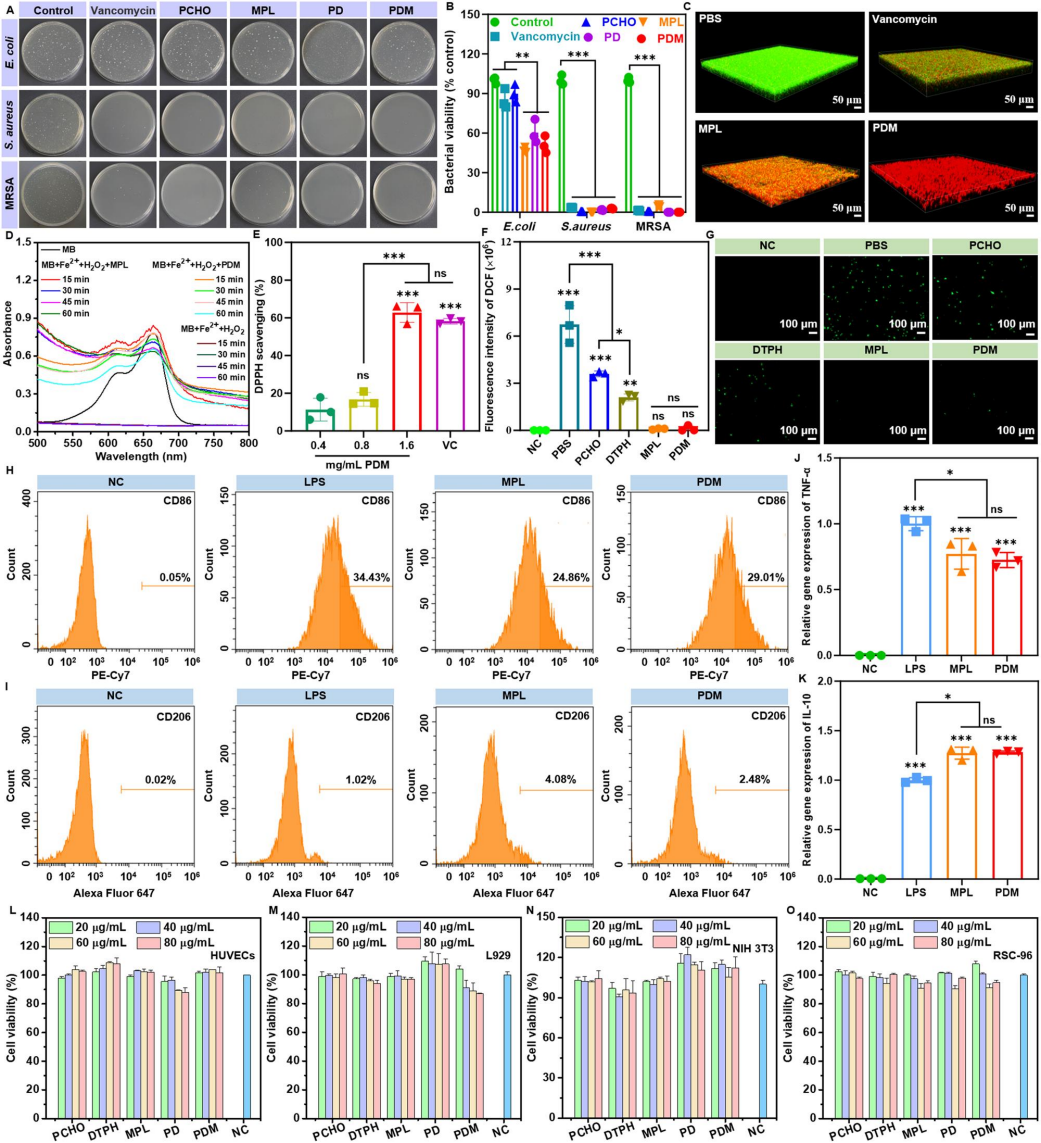
Antibacterial, antioxidant, anti-inflammatory activities and cytotoxicity assay *in vitro*. (**A**) Images of bacteria clones and (**B**) bacterial viability against *E. coli*, *S. aureus* and MRSA treated with different samples. (**C**) Confocal 3D images of MRSA biofilm after different treatments, scale bar: 50 μm. (**D**) Time-dependent UV-vis absorbance spectra of MB degradation with or without PDM treatment triggered by Fenton reaction induced by Fe^2+^ and H_2_O_2_. (**E**) DPPH scavenging percentage by PDM at different concentrations and VC. (**F**) Quantitative analysis of the fluorescence intensity of DCF in L929 cells after different treatments. (**G**) Images of ROS scavenging in L929 cells of different groups, ROS was labeled with DCFH-DA (scale bar: 100 μm). Quantitative analysis of (**H**) M1 macrophages and (**I**) M2 macrophages after treatment with PDM or controls by flow cytometry. (**J, K**) mRNA levels of TNF-α and IL-10 after treatment RAW 264.7 cells with MPL or PDM for 24 h. (**L–O**) Cell viability of PDM solution and each composite in HUVECs, L929, NIH3T3 and RSC-96 cells at different concentrations for 24 h (*P < 0.05, **P < 0.01, ***P < 0.001 and ns means not significant differences. *n* = 3).

The antibacterial effects of PDM hydrogel were evaluated against both Gram-negative bacteria, such as *E. coli*, and Gram-positive bacteria including methicillin-resistant *Staphylococcus aureus* (MRSA) and *S. aureus*, using a colony-counting assay. As shown in Figure 3A, untreated controls exhibited the highest bacterial counts across all strains. In contrast, bacterial viability was markedly reduced in the PCHO, MPL, PD and PDM groups, attributable in part to the excellent antibacterial contributions of pullulan and the cationic peptide EPL. PDM hydrogel achieved >99% inhibition of MRSA, which was resistant to multiply commonly used antibiotics. It also demonstrated 97% inhibition of *S. aureus*, and 48% inhibition of *E. coli*, outperforming vancomycin under identical conditions (Figure 3B). Confocal 3D imaging further revealed dense MRSA biofilms composed of viable bacteria in the PBS group, whereas almost no live biofilm was detected following PDM treatment (Figure 3C and Supplementary Figure S2A), demonstrating its potent antibiofilm capability. The antibacterial mechanism is related to the disruption of the integrity of bacterial cell membrane [41].

Given the critical role of oxidative stress in infected wound pathology [29, 46], the antioxidant capacity of PDM was assessed by hydroxyl radical (•OH) and 1,1-diphenyl-2-picrylhydrazyl (DPPH) scavenging assays [47]. Using MB as an indicator, both MPL and PDM significantly preserved MB absorbance during Fenton reactions compared with MB + Fe^2+^ + H_2_O_2_ controls (Figure 3D**)**. After 1 h, ∼82.2% of MB remained in PDM-treated group, indicating efficient •OH scavenging (Supplementary Figure S2B). Consistently, PDM exhibited concentration-dependent DPPH radical scavenging activity, reaching 62.8% at 1.6 mg/mL and exceeding that of vitamin C (56.9% at 2 mg/mL) (Figure 3E and Supplementary Figure S2C). Intracellular ROS scavenging was further examined in L929 cells exposed to H_2_O_2_. Strong DCF fluorescence was observed in PBS, PCHO and DTPH groups, indicating elevated oxidative stress, whereas MPL- and PDM-treated cells showed markedly reduced fluorescence, confirming effective intracellular ROS elimination (Figure 3F and G and Supplementary Figure S2D).

The anti-inflammatory effect of PDM was investigated in LPS-stimulated RAW 264.7 macrophages. Flow cytometric analysis revealed that LPS treatment induced a high proportion of M1 macrophages (CD86^+^, 34.43%), whereas MPL, PD and PDM treatments significantly reduced the M1 population to 24.86%, 28.09% and 29.01%, respectively (Figure 3H and Supplementary Figure S3A). Concurrently, the proportion of M2 macrophages (CD206^+^) increased in the MPL (4.08%), PD (1.5%) and PDM (2.48%) groups compared with negligible levels in the NC and LPS groups (Figure 3I and Supplementary Figure S3B), indicating macrophage polarization toward a regenerative phenotype. In comparison to the LPS group, the PD group exhibited a reduced M1 population and an elevated M2 population, suggesting that the hydrogel matrix also promoted M2 macrophage polarization. The PDM group demonstrated a higher M1 population and a lower M2 population compared to the MPL group, indicating that the hydrogel matrix slightly disrupted the M2 macrophage polarization effect of MPL *in vitro* under the complete solution state. Additionally, MPL and PDM treatments significantly downregulated TNF-α expression while upregulating the anti-inflammatory cytokine IL-10 at the mRNA level (Figure 3J and K). There was no significant difference between MPL and PDM groups in the expressions of TNF-α and IL-10, revealing that the PD did not interfere with the anti-inflammatory effect of MPL *in vitro*.

The cytotoxicity of PDM and control materials was assessed in HUVECs, L929, mouse embryonic fibroblast cell line (NIH 3T3) and rat Schwann cell-96 (RSC-96) cells over a concentration range of 20–80 μg/mL. As illustrated in Figure 3L–O, cell viabilities in all tested cell types remained above 85% even at the highest concentration, demonstrating the favorable biocompatibility of PDM and its components.

### Evaluation of autophagy, cell proliferation and migration *in vitro*

Autophagy is a tightly regulated intracellular self-degradation process that plays an essential role across multiple phases of wound healing, including inflammation resolution, proliferation and tissue remodeling [21, 22]. Controlled enhancement of autophagy facilitates efficient repair in chronic wounds [48]. Microtubule-associated proteins 1A/1B light chain 3A (LC3) is a core component of autophagosome formation, with cytosolic LC3-I converted to lipidated LC3-II during autophagosome maturation [49]. The autophagy receptor protein p62 mediates selective cargo delivery to autophagosomes and is degraded upon completion of autophagic flux [49]. Accordingly, LC3 conversion and p62 turnover are widely used indicators of autophagy activity and flux [50].

**Figure 4:**
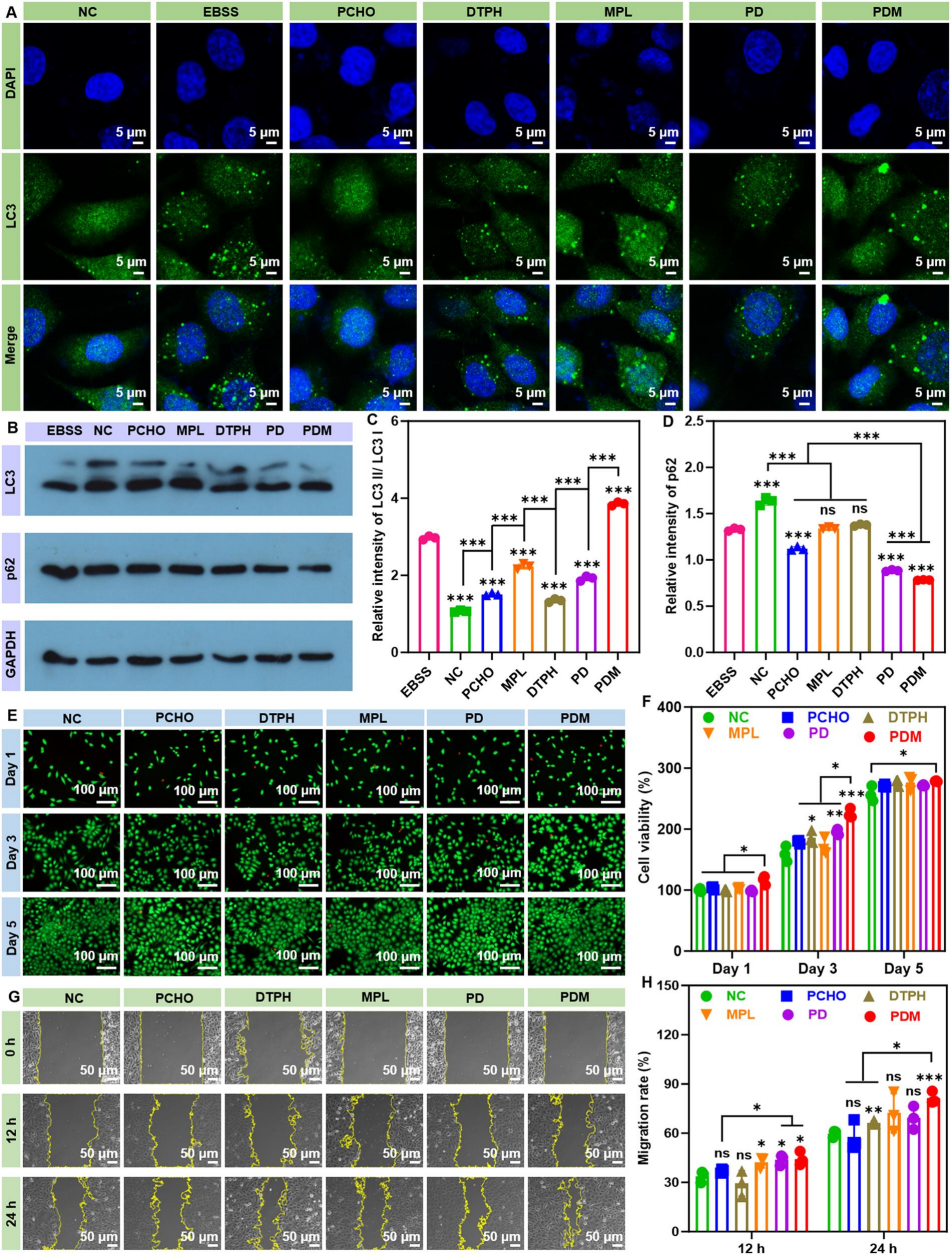
Evaluation of autophagy, cell proliferation and migration *in vitro*. (**A**) Immunofluorescence staining of LC3 in HUVECs after exposure to PDM solution and controls, scale bars are 5 μm. (**B**) Results from WB analyses of LC3 and p62 expression levels after treatment with PDM solution and controls for 24 h. (**C, D**) Semi-quantitative analyses of relative intensity of LC3Ⅱ/LC3I (C) and relative intensity of p62 (D) based on the WB results. (**E**) LIVE/DEAD staining images of L929 cells on Days 1, 3 and 5 after treatment with PDM solution and controls (20 μg/mL) (scale bar: 100 µm, *n* = 3). (**F**) The cell viability of L929 cells treated with PDM solution and controls on Days 1, 3 and 5. (**G**) Migration images of HUVECs after treatment with PDM solution and controls for 12 and 24 h, scale bars: 50 μm. (**H**) Quantitative analysis of migration rate of different groups using ImageJ software (*P < 0.05, **P < 0.01, ***P < 0.001 and ns means not significant differences. *n* = 3).

The effect of PDM on HUVECs' autophagy was analyzed using immunofluorescence staining and WB. As shown in Figure 4A, pronounced LC3 fluorescence was observed in the DTPH, MPL, PD and PDM groups, with higher signal intensity in the MPL and PDM groups, comparable to that of the positive control (Earle’s Balanced Salt Solution, EBSS) and exceeding that of the NC and PCHO groups. Western-blot analysis further demonstrated an increased LC3-II/LC3-I ratio in the MPL, PD and PDM groups relative to controls, indicating enhanced autophagosome formation (Figure 4B and C). In parallel, p62 was reduced following PDM treatment, consistent with intact autophagic flux rather than autophagosome accumulation (Figure 4B and D). These findings indicate that PDM effectively enhances autophagy activity in endothelial cells, a process closely associated with subsequent regenerative behaviors.

The influence of PDM on cell proliferation was next assessed using L929 fibroblasts. Cells were incubated with diluted PDM solution (20 μg/mL) or control formulations for up to five days. LIVE/DEAD staining revealed viable cells in all groups on Day 1, with progressively increased cell density and minimal cell death observed on Days 3 and 5, confirming good cytocompatibility (Figure 4E). Quantitative analysis using the Alamar Blue® assay showed that PDM-treated cells exhibited higher viability than all other groups on Day 1 (Figure 4F). While cell proliferation increased across all groups over time, the DTPH, PD and PDM groups displayed significantly higher viability than the NC group on Day 3. By Day 5, PDM group exhibited the highest cell viability, indicating a sustained pro-proliferative effect [51]. Although the precise molecular mechanisms remain to be fully elucidated, this effect may be related to activation of proliferation-associated signaling pathways, such as Wnt/β-catenin signaling, which are known to contribute to wound repair [3].

Cell migration, a critical step in re-epithelialization and angiogenesis, was evaluated using a scratch assay in HUVECs. After 12 h of incubation, all groups exhibited partial wound closure (Figure 4G). The migration rate of the PDM group (44.31%) was higher than that of the NC group (33.63%) and exceeded those of the PCHO and DTPH groups (Figure 4H). MPL and PD groups also showed enhanced migration compared with controls. After 24 h, migration rates further increased, with the PDM group achieving the highest wound closure (81.34%), compared with 59.72% in the NC group (Figure 4H). These results demonstrate that PDM promotes endothelial cell migration, a key process underlying angiogenesis and wound regeneration.

### Angiogenesis, gene expressions and Schwann cell maturation *in vitro*

**Figure 5:**
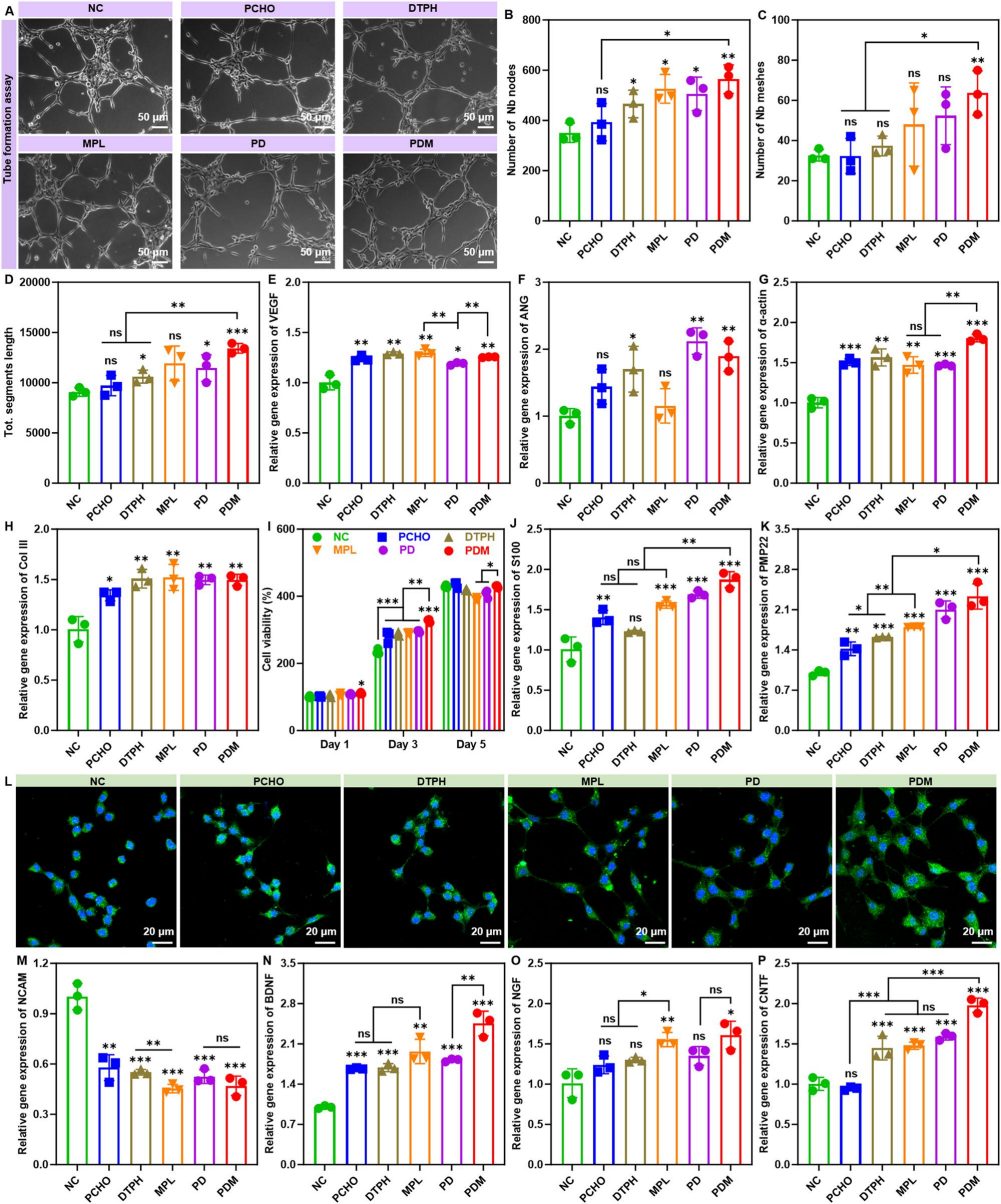
Angiogenesis, gene expressions and Schwann cell maturation assay *in vitro*. (**A**) Images of HUVECs tubule formation after PDM solution and controls treatment for 8 h, scale bars: 50 μm. Quantitative analysis of number of nodes (**B**), number of meshes (**C**) and total segments length (**D**). Cells without any treatment were used as NC. mRNA level of (**E**) VEGF, (**F**) ANG, (**G**) α-actin and (**H**) Col III after treatment HUVECs for 3 days. (**I**) Cell viability of RSC-96 cells treated with PDM and controls on Days 1, 3 and 5. (J, K) mRNA level of S100 (**J**) and *PMP22* (**K**) after treatment RSC-96 cells for 3 days. (**L**) The merged images of S100 immunofluorescence staining in RSC-96 cells after treatment with PDM or controls for 3 days (scale bar: 20 μm). mRNA level of (**M**) NCAM, (**N**) *BDNF*, (**O**) NGF and (**P**) *CNTF* after treatment RSC-96 cells with different samples for 3 days (*P < 0.05, **P < 0.01, ***P < 0.001, and ns means not significant differences. *n* = 3).

To further evaluate the proangiogenic potential of PDM *in vitro*, an endothelial tube formation assay was performed. As shown in Figure 5A, capillary-like structures were observed in all groups after 8 h of incubation. Quantitative analysis revealed that the number of nodes in the DTPH, MPL, PD and PDM groups were higher than those in the NC group (Figure 5B). The PDM group exhibited the greatest number of meshes and the longest total segment length among all groups (Figure 5C and D), indicating a pronounced enhancement of angiogenic network formation. Consistent with these observations, *qRT-PCR* analysis demonstrated upregulation of angiogenesis-related genes, including VEGF and *ANG*, in the PDM group relative to the NC group (Figure 5E and F). In addition, PDM treatment increased the mRNA expression levels of α-actin and Col III (Figure 5G and H), suggesting a supportive role in extracellular matrix deposition and tissue regeneration.

Schwann cells, the principal glial cells of the peripheral nervous system, play a critical role in wound repair by supporting nerve regeneration and modulating the local regenerative microenvironment [52, 53]. During maturation, Schwann cells acquire enhanced myelination capacity and secrete neurotrophic factors that promote axonal growth and tissue repair [45]. To assess the effect of PDM on Schwann cell behavior, RSC-96 cells were evaluated for proliferation, morphological maturation and gene expression. As shown in Figure 5I, PDM-treated cells exhibited higher viability than other groups on Day 1. Although robust proliferation was observed across all groups over time, the PDM group showed the highest cell viability on Day 3, indicating a transient pro-proliferative effect. By Day 5, no significant differences in cell viability were observed among most groups, with only a slight reduction detected in MPL group.

Gene expression analysis revealed that PDM upregulated myelination-associated markers, including S100 and peripheral myelin protein 22 (*PMP22*), with expression levels reaching 1.87- and 2.32-fold of NC, respectively (Figure 5J and K), indicative of accelerated Schwann cell maturation. Immunofluorescence staining of S100 further showed that Schwann cells in the NC group exhibited a rounded morphology, whereas cells in the MPL and PDM groups adopted an elongated, spindle-like morphology characteristic of mature Schwann cells (Figure 5L and Supplementary Figure S4). Moreover, neuronal cell adhesion molecule (NCAM), a marker highly expressed in immature Schwann cells, was downregulated in all treated groups, with the lowest expression observed in MPL and PDM groups (Figure 5M). Concurrently, neurotrophic factor genes, including brain-derived neurotrophic factor (*BDNF*), nerve growth factor (NGF) and ciliary neurotrophic factor (*CNTF*), were significantly upregulated in the PDM group compared with NC (Figure 5N–P), suggesting enhanced neurotrophic secretory function.

To explore the role of enhanced autophagy in facilitating angiogenesis and Schwann cell maturation by PDM, autophagy inhibition assays were carried out. As depicted in Supplementary Figure S5A, after Bafilomycin A1 (BafA1) treatment, capillary-like structures in the PDM+BafA1 group significantly decreased in comparison with the NC group. Quantitative analysis demonstrated that the numbers of nodes, meshes and total segment length in the PDM+BafA1 group were lower than those in the NC group (Supplementary Figure S5B–D), indicating a weakened angiogenic capacity. Moreover, the quantity of Schwann cells with an elongated, spindle-like morphology also declined in the PDM+BafA1 group, and a greater number of Schwann cells with a rounded morphology were observed, which was comparable to that of the NC group (Supplementary Figure S5E). These findings revealed that the blockade of autophagy reduces the proangiogenic or proneurogenic effects of PDM.

Collectively, these results demonstrate that PDM not only promotes angiogenesis but also facilitates Schwann cell maturation and neurotrophic factor production, thereby supporting reconstruction of a neurovascular regenerative microenvironment *in vitro*, which is closely associated with autophagy.

### *In vivo* anti-infection and MRSA-infected wound healing

**Figure 6:**
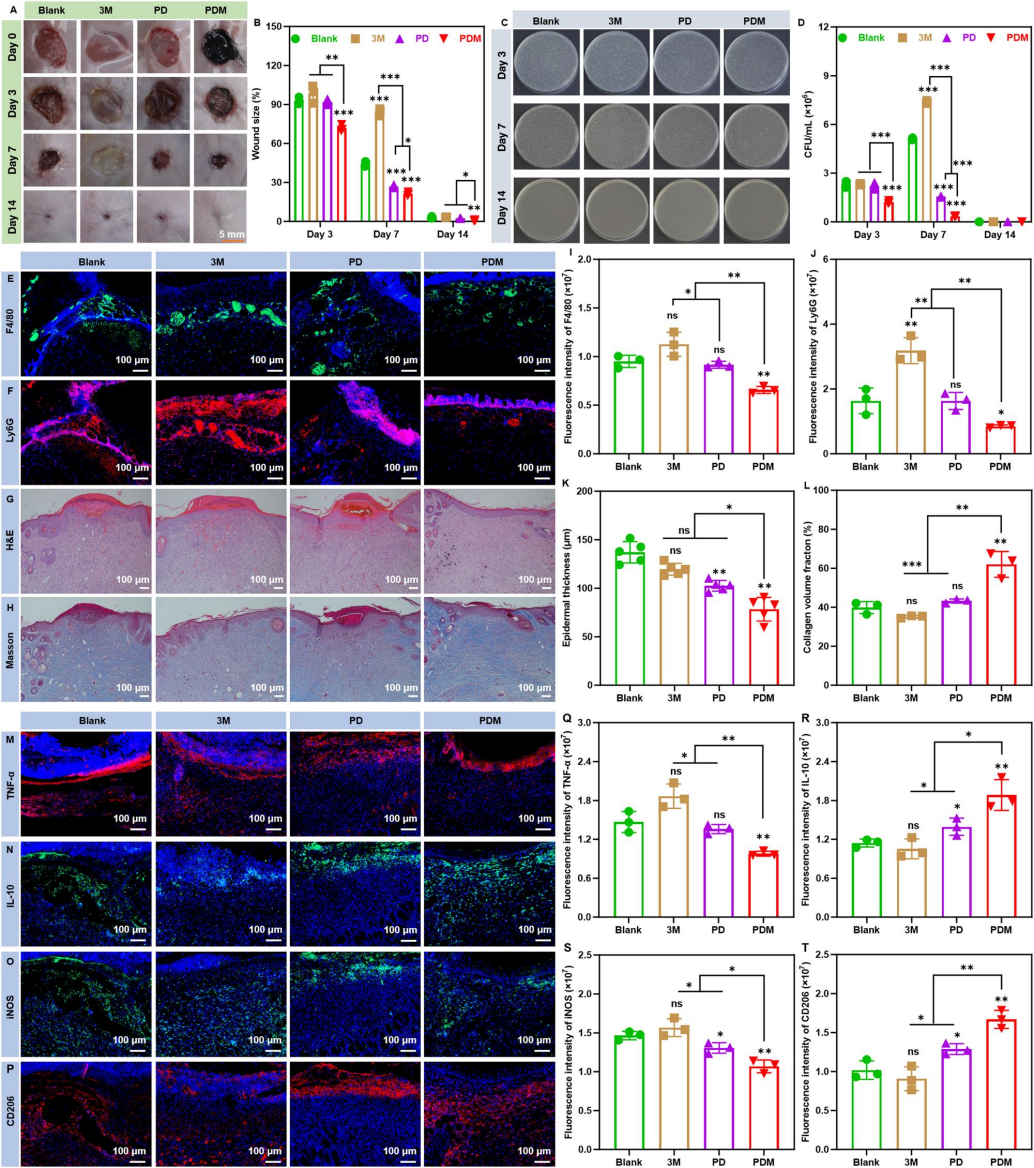
Therapeutic effects of PDM hydrogel on MRSA-infected wound healing *in vivo*. (**A**) Representative images of wounds at various time points after different treatments (scale bar: 5 mm). (**B**) Wound area size of PDM hydrogel and controls at different time points after the operation. (**C**) Images of MRSA colonies growing on the agar plates came from the homogenized infected tissues after various treatments on Days 3, 7 and 14. (**D**) Quantitative bacterial colonies densities based on (C). (**E, F**) Immunofluorescence staining of F4/80 and Ly6G from different groups on Day 3 (scale bar: 100 μm). (**G, H**) H&E and Masson’s trichrome staining of wound tissues on Day 14. (**I, J**) Quantitative analysis of the fluorescence intensity of F4/80 and Ly6G, respectively. (**K**) Epidermal thickness (*n* = 5) and (**L**) collagen content in wound tissues based on (G) and (H), respectively. (**M–P**) Immunofluorescence staining of TNF-α, IL-10, iNOS and CD206 from different groups on Day 7 (scale bar: 100 μm). (**Q–T**) Quantitative analysis of the fluorescence intensity of TNF-α, IL-10, iNOS and CD206, respectively (*P < 0.05, **P < 0.01, ***P < 0.001 and ns means not significant differences. *n* = 3).

Based on the *in vitro* findings, the therapeutic efficacy of PDM was further evaluated using a full-thickness MRSA-infected cutaneous wound model, as previously described [41]. Macroscopic imaging, histological analysis and immunofluorescence staining were performed on Days 3, 7 and 14 to assess wound healing progression. As shown in Figure 6A, all treatment groups except the 3M group exhibited visible wound contraction by Day 3. The PDM group displayed a smaller wound area, whereas the 3M group showed abundant purulent exudates indicative of severe infection. Quantitative analysis confirmed that the residual wound area in the PDM group (71.78%) was significantly lower than that in the Blank (92.82%), 3M (98.22%) and PD (91.70%) groups (Figure 6B). By Day 7, eschar formation was observed in all groups, accompanied by substantial wound closure in the Blank (44.67%) and PD (26.94%) groups. Importantly, the PDM group exhibited the smallest wound area (21.16%), whereas the 3M group showed minimal improvement (84.53%). By Day 14, although PD treatment modestly accelerated wound closure relative to Blank and 3M groups, residual eschar remained evident. In contrast, wounds treated with PDM were almost completely re-epithelialized, with a remaining wound area of only 0.8% and no visible eschar (Figure 6A and B), demonstrating the superior healing efficacy of PDM.

To assess *in vivo* antibacterial performance, wound tissues were harvested on Days 3, 7 and 14 and subjected to bacterial colony counting (Figure 6C and D). On Day 3, a large number of yellow secretions were observed at the wound site in the 3M group, indicating the successful establishment of MRSA-infected wound model (Figure 6A). Meanwhile, bacterial loads in the PDM group were lower than those in the Blank, 3M and PD groups, indicating effective early infection control. By Day 7, bacterial counts increased substantially in the Blank and 3M groups, particularly in the 3M group, reaching 7.37 × 10^6^ CFU mL^−1^, revealing an aggravation of infection. In contrast, bacterial burdens in the PDM and PD groups decreased to 0.35 × 10^6^ and 1.56 × 10^6^ CFU mL^−1^, respectively. By Day 14, bacterial counts declined in all groups, consistent with partial host clearance (Figure 6C and D). Immunofluorescence staining for F4/80 and Ly6G on Day 3 further corroborated these findings. Strong F4/80 and Ly6G signals were observed in the Blank and 3M groups, indicating pronounced macrophage and neutrophil infiltration, further confirming the feasibility of the animal modeling method. Moreover, on Day 3, the infection in these two groups has worsened. In contrast, PDM-treated wounds exhibited reduced fluorescence intensities, reflecting effective suppression of MRSA infection *in vivo* (Figure 6E, F, I and J and Supplementary Figure S6).

Histological evaluation of wound tissues was performed using H&E and Masson’s trichrome staining (Figure 6G and H and Supplementary Figure S7). On Day 3, scar tissue formation was evident in the Blank, PD and PDM groups, indicating initiation of repair, whereas the 3M group showed minimal tissue regeneration (Supplementary Figure S7A). By Day 7, newly formed tissue was apparent in the Blank, PD and PDM groups, with more pronounced regeneration in the PD and PDM groups. On Day 14, residual scar tissue persisted in the Blank, 3M and PD groups, whereas scar structures were largely absent in the PDM group, which exhibited the thinnest and most uniform epidermis (Figure 6G and K). Masson’s trichrome staining further revealed sparse and disorganized collagen deposition in the Blank and 3M groups during early stages, while PD treatment resulted in dense but irregular collagen accumulation. In contrast, PDM treatment promoted abundant, well-organized and highly aligned collagen deposition. By Day 14, PDM-treated wounds showed robust extracellular matrix reconstruction with minimal fibrotic architecture (Figure 6H and L and Supplementary Figure S7B), indicating reduced fibrosis and improved tissue quality.

To further characterize modulation of the wound microenvironment, inflammatory responses were evaluated by TNF-α and IL-10 staining on Day 7. Strong TNF-α signals were detected in the Blank and 3M groups, while the PD group exhibited moderate inflammation. In contrast, the PDM group displayed the lowest TNF-α expression (Figure 6M and Q and Supplementary Figure S8A) and the highest IL-10 levels (Figure 6N and R and Supplementary Figure S8B), indicating effective inflammation resolution. Given the critical role of macrophage polarization in wound repair [51, 54], M1 and M2 macrophage populations were further assessed using iNOS and CD206 staining. Abundant iNOS^+^ macrophages were observed in the Blank and 3M groups, whereas PD treatment moderately reduced M1 polarization, further suggesting that the hydrogel matrix also possessed immunomodulatory properties *in vivo* as shown *in vitro* assay. Notably, PDM treatment resulted in the lowest iNOS signal and a marked increase in CD206^+^ macrophages (Figure 6O, P, S and T and Supplementary Figure S8C and D), demonstrating effective reprogramming of macrophages toward a pro-regenerative phenotype. The disparity in the immunomodulation effects of PDM *in vitro* and *in vivo* is probably attributed to the fact that the *in vitro* assay conditions failed to mirror the sustained release kinetics. Alternatively, it was possible that the immunomodulatory mechanism *in vivo* operated via alternative pathways that were not captured by the LPS-stimulated RAW 264.7 model.

Collectively, these *in vivo* results demonstrate that PDM hydrogel effectively suppresses MRSA infection, accelerates wound closure, reduces fibrosis and orchestrates immune microenvironment remodeling, thereby enabling efficient and high-quality healing of MRSA-infected wounds.

**Figure 7:**
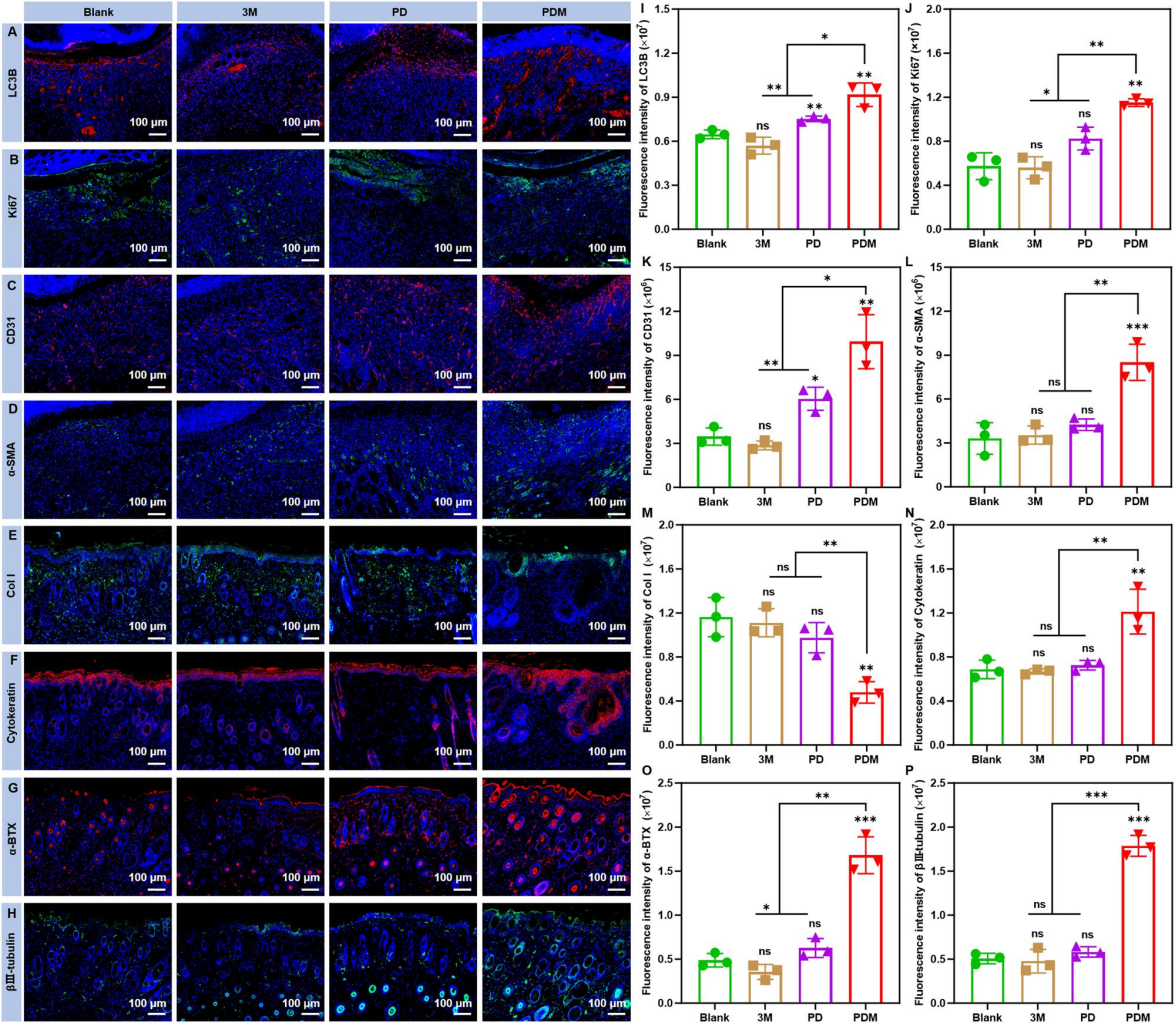
Effects of PDM hydrogel on the *in vivo* repair process. (**A–D**) Immunofluorescence staining of LC3B, Ki67, CD31 and α-SMA from different groups on Day 7 (scale bar: 100 μm). (**E–H**) Immunofluorescence staining of Col I, Cytokeratin, α-BTX and βIII-tubulin from different groups on Day 14 (scale bar: 100 μm). (**I–P**) Quantitative analysis of the fluorescence intensity of LC3B, Ki67, CD31, α-SMA, Col I, Cytokeratin, α-BTX and βIII-tubulin, respectively (*P < 0.05, **P < 0.01, ***P < 0.001 and ns means not significant differences. *n* = 3).

Additionally, immunofluorescence staining for LC3B, Ki67, CD31 and α-smooth muscle actin (α-SMA) was performed on Day 7 to evaluate autophagy, cell proliferation and angiogenesis within the wound region, respectively [55]. As depicted in Figure 7A, the PD group showed increased LC3B expression compared with the Blank and 3M groups. The PDM group displayed the strongest LC3B fluorescence, with an intensity ∼1.4-fold higher than that of the Blank group (Figure 7A and I and Supplementary Figure S9A), indicating enhanced autophagic activity *in vivo*. Consistently, Ki67-positive staining was observed in all groups, whereas the PDM group exhibited the highest Ki67 signal intensity (Figure 7B and J and Supplementary Figure S9B), suggesting robust cell proliferation in the wound area. Immunofluorescence analysis of CD31 revealed weak vascular signals in the Blank and 3M groups, moderate staining in the PD group and the strongest expression in the PDM group (Figure 7C and K and Supplementary Figure S9C), indicative of enhanced neovascularization. This observation was consistent with the *in vitro* tube formation results. Furthermore, α-SMA staining, a marker of vascular maturation, was increased in the PDM group, whereas relatively weak signals were detected in the Blank, 3M and PD groups (Figure 7D and L and Supplementary Figure S9D), suggesting that PDM promotes maturation of newly formed blood vessels. Collectively, these results demonstrate that PDM enhances autophagy, cell proliferation and angiogenic remodeling during wound healing.

To further assess healing quality during the remodeling stage, collagen deposition, re-epithelialization and cutaneous innervation were examined by immunofluorescence staining on Day 14. Type I collagen (Col I) staining revealed lower fluorescence intensity in the PDM group compared with the other groups (Figure 7E and M and Supplementary Figure S10A), indicating reduced fibrosis and scar formation, consistent with histological findings [56]. Re-epithelialization was evaluated by cytokeratin staining, which was detected in all groups. Notably, the PDM group exhibited stronger cytokeratin expression than the Blank, 3M and PD groups (Figure 7F and N and Supplementary Figure S10B), indicating accelerated epidermal regeneration. No significant differences in Col I or cytokeratin expression were observed among the Blank, 3M and PD groups.

To explore potential regulatory mechanisms, α-BTX staining was performed to label α7-nAChR. Minimal α-BTX fluorescence was observed in the Blank, 3M and PD groups, whereas the PDM group exhibited elevated α7-nAChR expression, ∼3.45-fold higher than that of the Blank group (Figure 7G and O and Supplementary Figure S10C). These findings suggest that PDM may enhance autophagy and α7-nAChR signaling [57], thereby suppressing excessive inflammation while promoting angiogenesis and re-epithelialization. Finally, cutaneous innervation was assessed by immunofluorescence staining for βIII-tubulin [53, 58]. In the PDM group, abundant βIII-tubulin-positive fibers were observed throughout both the epidermis and dermis, whereas only sparse signals were detected in the other groups (Figure 7H). Quantitative analysis showed that βIII-tubulin fluorescence intensity in the PDM group was 3.53-, 3.75- and 3.38-fold higher than that in the Blank, 3M and PD groups, respectively (Figure 7P and Supplementary Figure S10C), indicating markedly enhanced nerve regeneration.

Taken together, these findings demonstrate that PDM hydrogel promotes high-quality healing of MRSA-infected wounds by suppressing infection and inflammation, promoting M2 macrophage polarization, enhancing autophagy, stimulating cell proliferation and angiogenesis, reducing fibrosis, accelerating re-epithelialization and restoring cutaneous innervation.

### Mechanism underlying PDM-accelerated MRSA-infected wound healing

**Figure 8:**
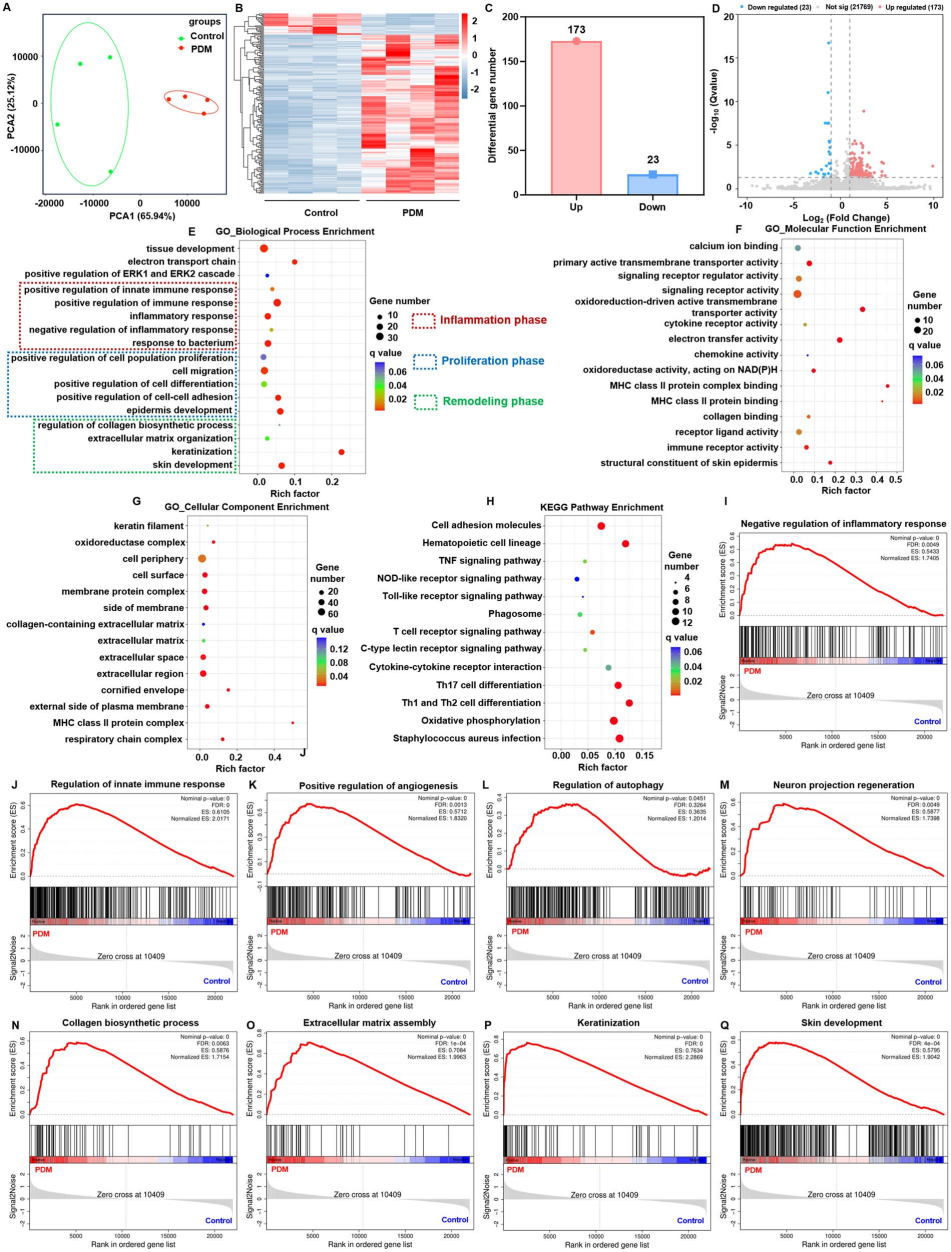
Transcriptomic analysis of MRSA-infected wound tissues on 7 days after surgery (*n* = 4). (**A**) PCA of the transcriptomic data. (**B**) Heatmap of significantly upregulated and downregulated genes after PDM hydrogel treatment in comparison with the control group. (**C**) Histogram showing the number of upregulated and downregulated genes in the PDM group. (**D**) Volcano plot of differentially expressed genes in PDM group in comparison with the control group. GO enrichment analysis of (**E**) biological process, (**F**) molecular function and (**G**) cellular component for differentially expressed genes in the PDM group. (**H**) KEGG enrichment analysis of differentially expressed genes in the PDM group. GSEA of the (**I**) negative regulation of inflammatory response, (**J**) regulation of innate immune response, (**K**) positive regulation of angiogenesis, (**L**) regulation of autophagy, (**M**) neuron projection regeneration, (**N**) collagen biosynthetic process, (**O**) extracellular matrix assembly, (**P**) keratinization and (**Q**) skin development.

To elucidate the molecular mechanisms by which PDM hydrogel accelerates MRSA-infected wound healing, RNA sequencing (RNA-seq) was performed on wound tissues harvested from the PDM-treated and Blank control groups. Principal component analysis (PCA) demonstrated high reproducibility and clear separation between the two groups, indicating distinct transcriptional profiles induced by PDM treatment (Figure 8A). Heatmap analysis further revealed robust differential gene expression between control and PDM-treated wounds (Figure 8B). Compared with the Blank group, 173 genes were upregulated and 23 genes were downregulated in the PDM group (Figure 8C and D), suggesting that PDM induces broad transcriptional reprogramming associated with wound repair.

Gene ontology (GO) enrichment analysis revealed that differentially expressed genes were predominantly associated with biological processes related to immune regulation, inflammatory response, cell proliferation and migration, epidermal development, collagen biosynthesis, extracellular matrix organization, keratinization and skin development (Figure 8E). These findings indicate that PDM simultaneously modulates immune responses and regenerative processes essential for effective wound healing. Consistent with this, molecular function analysis showed enrichment in signaling receptor activity, cytokine receptor activity, collagen binding, immune receptor activity and structural constituents of the epidermis, while cellular component analysis highlighted enrichment in keratin filaments, cell membranes and extracellular matrix components (Figure 8F and G).

Kyoto Encyclopedia of Genes and Genomes (KEGG) pathway analysis identified 13 significantly enriched pathways, including cell adhesion molecules, TNF signaling, NOD-like receptor signaling, Toll-like receptor signaling, phagosome formation, T cell receptor signaling, cytokine–cytokine receptor interaction and Th17 cell differentiation (Figure 8H). Among these, the most relevant pathways encompass cell adhesion molecules, TNF signaling pathway, phagosome, lysosome and hematopoietic cell lineage. These pathways collectively reflect coordinated regulation of host defense, inflammation resolution, autophagy and tissue regeneration during MRSA-infected wound healing. Furthermore, gene set enrichment analysis (GSEA) demonstrated that PDM treatment markedly suppressed inflammatory response pathways while enhancing pathways associated with innate immune regulation, angiogenesis, autophagy, neuronal projection regeneration, collagen biosynthesis, extracellular matrix assembly, keratinization and skin development (Figure 8I–Q).

Collectively, these transcriptomic analyses reveal that PDM hydrogel promotes MRSA-infected wound healing through integrated modulation of immune responses, autophagy activation, neurovascular regeneration, extracellular matrix remodeling and epidermal reconstruction, providing mechanistic support for the observed *in vivo* therapeutic outcomes.

Achieving complete and functional regeneration of MRSA-infected wounds remains a major clinical challenge [59, 60]. Persistent bacterial colonization sustains inflammatory signaling and disrupts the local wound microenvironment, thereby impairing coordinated tissue repair [61, 62]. Emerging evidence further indicates that autophagy plays a central regulatory role across multiple stages of wound healing by integrating immune resolution, cellular metabolism, angiogenesis and tissue remodeling [22]. Accordingly, therapeutic strategies capable of simultaneously controlling infection, reprogramming the immune microenvironment and restoring autophagic balance are highly desirable for accelerating MRSA-infected wound repair.

MXene-based materials have attracted increasing interest owing to their favorable biocompatibility, antioxidant and anti-inflammatory properties, and emerging roles in autophagy regulation [32, 35]. However, the intrinsic limitations of MXene nanosheets—including susceptibility to oxidation, interlayer aggregation and poor tissue retention—have restricted their direct biomedical application. In this work, assembly of Ti_3_C_2_Tₓ MXene nanosheets into flower-shaped microspheres and subsequent functionalization with EPL effectively addressed these constraints while enhancing antibacterial performance. The flower-shaped MPL provide notable advantages for wound healing, including reduced restacking, enhanced biocompatibility, improved stability within the moist wound microenvironment, and better integration into dressing matrix. Collectively, these features contribute to sustained antibacterial, antioxidant, anti-inflammatory and autophagy-regulating functions, as well as an increase in cell-supportive properties. Nevertheless, the solution-state nature and limited tissue adhesion of MPL alone hinder its direct use for wound repair. To overcome these challenges, a multifunctional hydrogel platform (PDM) was rationally designed by integrating MPL into a dynamic PCHO–DTPH network, thereby enabling localized retention, structural stability and sustained bioactivity at the wound site.

From a materials design perspective, multifunctionality is critical for infected wound dressings, which must simultaneously provide antimicrobial activity, bioactivity and appropriate mechanical and interfacial properties [63, 64]. The dynamic Schiff-base chemistry of PDM confers pH/redox responsiveness, self-healing behavior and robust tissue adhesion, supporting hemostasis and stable wound coverage while adapting to the pathological microenvironment [65–67]. Importantly, incorporation of MPL not only reinforces the hydrogel network but also introduces electrical conductivity, which is closely related to neurovascular regeneration through bioelectric signal modulation [33, 34]. Moreover, the conductivity may also enhance endogenous electrical signaling at the wound interface, which has been associated with enhanced cell migration, proliferation and tissue regeneration. In addition, the antibacterial activity plays a significant role in minimizing the risk of wound infection, which is crucial since a persistent microbial load can prolong inflammation and impede tissue repair. MPL contributes to effective disruption of MRSA biofilms, which are highly resistant to immune clearance and antimicrobial therapies and represent a major obstacle to infected wound healing [64]. The ability of PDM to overcome biofilm-associated resistance likely reflects synergistic physical interactions and microenvironmental modulation, consistent with prior observations in biofilm-disruptive materials [68].

Beyond infection control, successful wound healing requires resolution of oxidative stress and inflammation, restoration of autophagic homeostasis and coordinated tissue regeneration. The antioxidant function can assist in scavenging excessive ROS at the wound area, thereby mitigating oxidative stress and protecting resident cells, newly formed tissues and extracellular matrix components from impairment. The immunomodulatory function probably plays a role in balancing the inflammatory response, suppressing excessive inflammation and facilitating a more favorable microenvironment for wound repair. By integrating antioxidant, immunomodulatory and autophagy-regulating functions within a single platform, PDM establishes a regenerative microenvironment that supports fibroblast proliferation, endothelial migration, angiogenesis and extracellular matrix remodeling [21, 22]. Notably, Schwann cell maturation and enhanced neurotrophic factor expression further contribute to reconstruction of neurogenic niche, which is increasingly recognized as a determinant of high-quality and functional wound repair [53]. The combined *in vitro* and *in vivo* findings indicate that PDM orchestrates immune regulation, autophagy enhancement, fibrosis reduction and cutaneous reinnervation in a synergistic manner, thereby addressing multiple pathological barriers associated with MRSA-infected wounds. The superior performance of PDM relative to PD underscores the pivotal contribution of MPL, particularly in nerve regeneration and functional tissue restoration.

In the broader context of infected wound therapies, antibacterial and bioactive hydrogels represent a promising alternative to conventional antibiotic-based approaches. Compared with existing strategies such as peptide hydrogels, extracellular matrix-mimetic materials, metal-phenolic networks, polymeric nanofibers or traditional dressings [64, 69–72], the PDM hydrogel promotes efficient MRSA-infected wound healing without reliance on antibiotics, exogenous growth factors or cell transplantation. This simplified and integrative approach may reduce treatment complexity and associated healthcare costs while minimizing risks linked to antimicrobial resistance. Despite these encouraging findings, further investigation is warranted to evaluate therapeutic efficacy in large-animal models. The immunomodulatory mechanisms of PDM both *in vivo* and *in vitro* require further elucidation. Such study will be essential for advancing PDM toward clinical translation and validating its potential as an antibiotic-free, multifunctional platform for treating MRSA-infected wounds in humans.

## Conclusion

In summary, we developed an antibacterial, conductive and bioactive flower-shaped MXene microsphere-based hydrogel that integrates multifunctional MPL within a dynamically crosslinked, pH/redox-responsive matrix for targeted MRSA-infected wound treatment. The PDM hydrogel combines rapid hemostasis with antioxidant, anti-inflammatory and antibiofilm activities, enabling effective infection control and microenvironment remodeling. Through coordinated immunomodulation and autophagy regulation, PDM promotes M2 macrophage polarization, cell proliferation, migration, angiogenesis and neural regeneration. Notably, a single application markedly accelerated healing of full-thickness MRSA-infected wounds, enhanced cutaneous innervation and reduced fibrosis *in vivo* with negligible cytotoxicity. These results underscore the pivotal role of MPL in structural stabilization and neurovascular repair. Collectively, PDM represents a promising antibiotic-free hydrogel platform with translational potential for clinical management of MRSA-infected and other complex infected wounds.

## Supplementary Material

rbag108_Supplementary_Data
