# Supplementary material for: A conductive MXene hydrogel reprograms immunity and autophagy to restore neurovascular repair in infected wounds
Source: Regen Biomater. 2026 Jun 3;13:rbag108. doi: 10.1093/rb/rbag108 (PMC13330540; doi:10.1093/rb/rbag108)
Supplement: rbag108_Supplementary_Data [file rbag108_supplementary_data.zip › 09-Jun-2026_013710_Supporting_information-Final_version.docx]

**Supporting Information**

**A conductive MXene hydrogel reprograms immunity and autophagy to restore neurovascular repair in infected wounds**

Yuanhui Xiao ^1^*^,#^*, Qingting Wu ^1^*^,#^*, Chunping Zeng ^1^*^,#^*, Yanyou Li ^1^, Kai Wang ^1^, Ziyu Peng ^1^, Yuner Luo ^2^, Zixi Wang ^1^, Lichun Huang ^1^, Shanshan Huang ^1^, Daolin Tang ^5^, Baoliang Zhang ^3,*^, Qiuyu Zhang ^3^*^,*^*, Jinbao Liu ^4,*^, Li Zhou ^1,*^

^1^ Guangdong Provincial Key Laboratory of Protein Modification and Disease, Key Laboratory of Biological Targeting Diagnosis, Therapy and Rehabilitation of Guangdong Higher Education Institutes, School of Basic Medical Sciences and The Fifth Affiliated Hospital, Guangzhou Medical University, Guangzhou, 511436, P. R. China.

^2^ Nanshan School, Guangzhou Medical University, Guangzhou, 511436, P. R. China.

^3^ Key Laboratory of Special Functional and Smart Polymer Materials of Ministry of Industry and Information Technology, School of Chemistry and Chemical Engineering, Northwestern Polytechnical University, Xi'an 710129, P. R. China.

^4^ Affiliated Cancer Hospital & Institute of Guangzhou Medical University, Guangzhou Municipal and Guangdong Provincial Key Laboratory of Protein Modification and Disease, State Key Laboratory of Respiratory Disease, School of Basic Medical Sciences, Guangzhou Medical University, Guangzhou, 511436, P. R. China.

^5^ Department of Surgery, UT Southwestern Medical Center, Dallas, TX 75390, USA

^*^ Correspondence: [blzhang@nwpu.edu.cn](mailto:blzhang@nwpu.edu.cn) (B.Z.); [qyzhang@nwpu.edu.cn](mailto:qyzhang@nwpu.edu.cn) (Q.Z.); [jliu@gzhmu.edu.cn](mailto:jliu@gzhmu.edu.cn) (J.L.); [zhouli@gzhmu.edu.cn](mailto:zhouli@gzhmu.edu.cn) (L.Z.).

^#^ These authors contributed equally to this article.

**Experimental section**

**1.1 Materials**

Pullulan, Sodium periodate (NaIO_4_), 3,3'-dithiobis(propionohydrazide) (DTPH), Hydrogen peroxide (H_2_O_2_), Dithiothreitol (DTT), Lipopolysaccharide (LPS) and LiF/HCl solution were purchased from Sigma-Aldrich (St. Louis, MO, USA). Ti_3_AlC_2_ was obtained from Forsman Scientific Co., Ltd (Beijing, China). ε-Poly-L-lysine (EPL, Mn = 3500 Da) was purchased from Zhengzhou Bainafo Bioengineering Co., Ltd. (Henan, China). Phosphate buffered saline (PBS), Fetal bovine serum (FBS), Dulbecco's modified Eagle's medium (DMEM) and LIVE/DEAD baclight viability/cytotoxicity kit were obtained from GIBCO, life technologies. All other chemicals were used as received without further treatment. Mouse fibroblasts cells (L929, RRID: CVCL_AR58), Mouse embryonic fibroblasts (NIH 3T3, RRID: CVCL_0594), Human umbilical vein endothelial cells (HUVECs, RRID: CVCL_2959), Mouse monocytic macrophage leukemia cells (RAW 264.7, RRID: CVCL_0493) and rat Schwann cell-96 (RSC-96, RRID: CVCL_4694) cells were provided by the National Collection of Authenticated Cell Cultures. The above cells were cultured in DMEM medium containing 10% FBS at 37℃ and 5% CO_2_.

**1.2 Synthesis of Ti_3_C_2_T_x_ MXene and** **Flower-shaped MPL microspheres**

The Ti_3_C_2_T_x_ MXene nanosheets was prepared using a similar method as described previously [[1](#_ENREF_1)]. Briefly, 1 g of Ti_3_AlC_2_ was added to the LiF/HCl solution and stirred at 35°C for 24 h. The suspension was centrifuged and washed repeatedly with deionized water until pH was about 7. The product underwent‌ ultrasonic treatment for 15 minutes, during which fragments and incompletely stripped substances were removed through centrifugation. Deionized water was then added to the dark green product, and it was ultrasonically treated to obtain a dispersion of stripped Ti_3_C_2_T_x_ MXene nanosheets.

Flower-shaped Ti_3_C_2_T_x_ MXene micropheres were prepared by ultrasonic spray [[2](#_ENREF_2)]. Typically, the Ti_3_C_2_T_x_ MXene nanosheets dispersion with a concentration of 4 mg/mL, was injected into the ultrasonic nozzle at a flow rate of 0.5 mL/min. The ultrasonic power on the nozzle was set to 1.5 W. The liquid droplets produced by ultrasonic atomization were sprayed into liquid paraffin at 150^o^C, where the water evaporated under magnetic stirring. After the reaction, flower-shaped microspheres were obtained through centrifugation, cleaning and freeze-drying.

EPL-modified flower-shaped MXene microspheres was formed by electrostatic adsorption. Briefly, 0.5 g Ti_3_C_2_T_x_ MXene flower-shaped microspheres were ultrasonically dispersed in a mixture of 60 mL ethanol and water with an alcohol-to-water ratio of 1:4. Subsequently, 1.2 g EPL were added and the mixture was mechanically stirred at 30°C for 24 h. Following centrifugation, water washing and freeze-drying, EPL-modified flower-shaped Ti_3_C_2_T_x_ MXene microspheres (MPL) were obtained.

**1.3 Characterizations of PDM hydrogel and controls**

Fourier transform infrared spectroscopy (FTIR) of flower-shaped Ti_3_C_2_T_x_ MXene micropheres, MPL, PCHO and PDM were recorded with a FTIR spectrometer in the range between 4000 and 400 cm^-1^ (Nicolet 6700, Thermo). The structure and elemental mapping of flower-shaped Ti_3_C_2_T_x_ MXene micropheres and MPL were examined by transmission electron microscopy (TEM, Talos F200X, FEI) equipped with an energy-dispersive spectrometer (EDS) at an accelerating voltage of 100 kV. The morphologies of PCHO, PD and PDM were observed by scanning electron microscope (Verios G4, FEI) after sprayed with a thin gold layer.

**1.4 Evaluation of multifunctional properties**

The pH/redox-responsiveness of PDM was analyzed *via* macroscopic assay [[3](#_ENREF_3)]. Briefly, 40 μL diluted HCl solution was added to PDM (200 μL), PDM was transformed from a gel state to a solution state. Additionally, 30 μL DTT solution (5%) and 30 μL H_2_O_2_ (15%) were successively added to PDM, respectively. Then, the gel-sol-gel process of PDM was observed. The states of PDM hydrogel were captured by digital photographs.

The self-healing capability of PDM was investigated by a macroscopic assay as reported previously [[4](#_ENREF_4)]. Briefly, PDM hydrogel was divided into two sections. Subsequently, these two sections were reassembled for approximately 1 minute and the hydrogel state was observed and documented with digital photographs. Additionally, the stability of PDM hydrogel was evaluated by capturing images to record the state changes of PDM following its placement at room temperature.

The conductivity of PDM hydrogel was determined by the four-probe method [[5](#_ENREF_5)]. In brief, a 100 μL samples was prepared on the coverslips, subsequently, the sheet resistance (R_S_) and thickness (t) of the samples were tested by a digital four-probe tester equipped with a linear probe head (1.0 mm spacing, 1 mA, Agilent Model B2900A) and a dial indicator, respectively. The conductivity (σ) was calculated according to the following equation: $\text{σ = }\frac{\text{1}}{\text{R}_{\text{s}}\text{t}}$ (Equation S1). Additionally, whether the current path consisting of PDM hydrogel and wires could cause the diode to emit light was also used to test the PDM’s conductivity.

The fresh porcine skin was also utilized to assess the adhesive strength of PDM hydrogel to the host tissue, as previously described [[4](#_ENREF_4)]. In brief, two pieces of skin (each measuring 10 mm × 10 mm) were secured onto the parallel plates of the rheometer, maintaining a gap of 500 μm. Then, 100 μL of PDM or controls were introduced into the gap between the two porcine skins. Then, the skins were pressed at 37^o^C for 2 min, and the normal stress was measured using a TA rheometer (DHR-2).

The hemostatic property of PDM was evaluated using a mouse hemorrhaging liver model (Kunming mice, 20-30 g, male). Briefly, the mice were anesthetized with isoflurane, fixed, and then the liver was exposed and placed on pre-weighed filter paper (W_0_). The tissue fluid surrounding the liver was carefully removed, and the liver was punctured with an 18 G needle to induce bleeding. Subsequently, 50 μL of PDM was applied to the bleeding site, and the hemorrhaging site was photographed at specific intervals of 0, 30, 45 and 60 seconds. After 60 seconds, the filter paper (W_t_) with the absorbed blood was weighed. The blood loss in the mice was equivalent to the increase in the weight of the filter paper (W_t_ - W_0_). No treated mice were utilized as a control.

*E. coli*, *S. aureus* and *MRSA* (methicillin-resistant *Staphylococcus aureus*) were employed to assess the antibacterial capacity of PDM hydrogel. Specifically, 10 μL of bacterial suspensions (with a concentration of 10⁶ CFU/mL) were added to the surface of 400 μL PDM hydrogel in a 24-well cell plate. PCHO, MPL, and PD (without MPL) were utilized as control groups. Moreover, vancomycin was used as a positive control. Following a 2-hour incubation period, the bacteria were detached from the surface using 990 μL of PBS, diluted to an appropriate concentration, spread on a solid agar plate, and cultured for an additional 18 hours to enumerate the colony-forming units (CFU). The bacterial viability=$\frac{{CFU}_{experimental group}}{{CFU}_{control group}}\times100\%$ (Equation S2).

**1.5 *qRT-PCR* analysis *in vitro***

The gene expression levels of TNF-α, IL-10, VEGF, *ANG*, α-actin, Col III, S100, *PMP22*, NCAM, *BDNF*, NGF and *CNTF* after cells treated with diluted PDM solution (final concentration 20 μg/mL) were evaluated by *qRT-PCR*, respectively. Total RNA of RAW 264.7, HUVECs, NIH 3T3 or RSC-96 cells were isolated and the cDNA synthesis was performed by Transcriptor First Strand cDNA Synthesis Kit (Abm). *qRT-PCR* using SYBR Green Premix Pro Tan HS *qPCR* Kit was conducted by a PCR Amplifier (Gentier 96R). The primer sequences as following:

| Gene | Sequences |
| --- | --- |
| TNF-α | Forward: CCCTCACACTCAGATCATCTTCT  Backward: GCTACGACGTGGGCTACAG |
| IL-10 | Forward: GCTCTTACTGACTGGCATGAG  Backward: CGCAGCTCTAGGAGCATGTG |
| VEGF | Forward: GAGATGTCCCTGGAAGAACACA  Backward: GAGTGGGATGGGTGATGTCAG |
| *ANG* | Forward: AGAAGCGGGTGAGAAACAAAAC  Backward: AGTGCTGGGTCAGGAAGTGTG |
| α-actin | Forward: ATCTGGCACCACACCTTCTA  Backward: AGCTCGTAGCTCTTCTCCAG |
| Collagen Ⅲ | Forward: GCTGGCATTCCTCAGACTTC  Backward: TAGTCTCATTGCCTTGCGTG |
| S100-Rat | Forward: GTTGCCCTCATTGATGTCT  Backward: CTGCTCTTTGATTTCCTCC |
| *PMP22*-Rat | Forward: TGTACCACATCCGCCTTGG  Backward: GAGCTGGCAGAAGAACAGGAAC |
| NCAM-Rat | Forward: TTCAGTGACGACAGTTCGGAGC  Backward: TGCGAAGACCTTGAGGTGGAT |
| *BDNF*-Rat | Forward: TCTACGAGACCAAGTGTAATCC  Backward: TTATGAACCGCCAGCCAAT |
| NGF-Rat | Forward: CGCTCTCCTTCACAGAGTTTT  Backward: GACATTACGCTATGCACCTCAGA |
| *CNTF*-Rat | Forward: ATGGCTTTCGCAGAGCAAAC  Backward: CAACGATCAGTGCTTGCCAC |
| hGAPDH | Forward: GGAGCGAGATCCCTCCAAAAT  Backward: GGCTGTTGTCATACTTCTCATGG |
| mGAPDH | Forward: CATCACTGCCACCCAGAAGACTG  Backward: ATGCCAGTGAGCTTCCCGTTCAG |

**Table**

**Table S1. Final formulation of hydrogel.**

|  | PCHO | DTPH | MPL | H_2_O |
| --- | --- | --- | --- | --- |
|  | (10 wt%) | (2.5 wt%) | (5 wt%) |  |
| PDM | 90 μL | 5 μL | 5 μL | --- |
| PD | 90 μL | 5 μL | --- | 5 μL |
| PCHO | 90 μL | --- | --- | 10 μL |

**Figures**

**Figure S1**

**
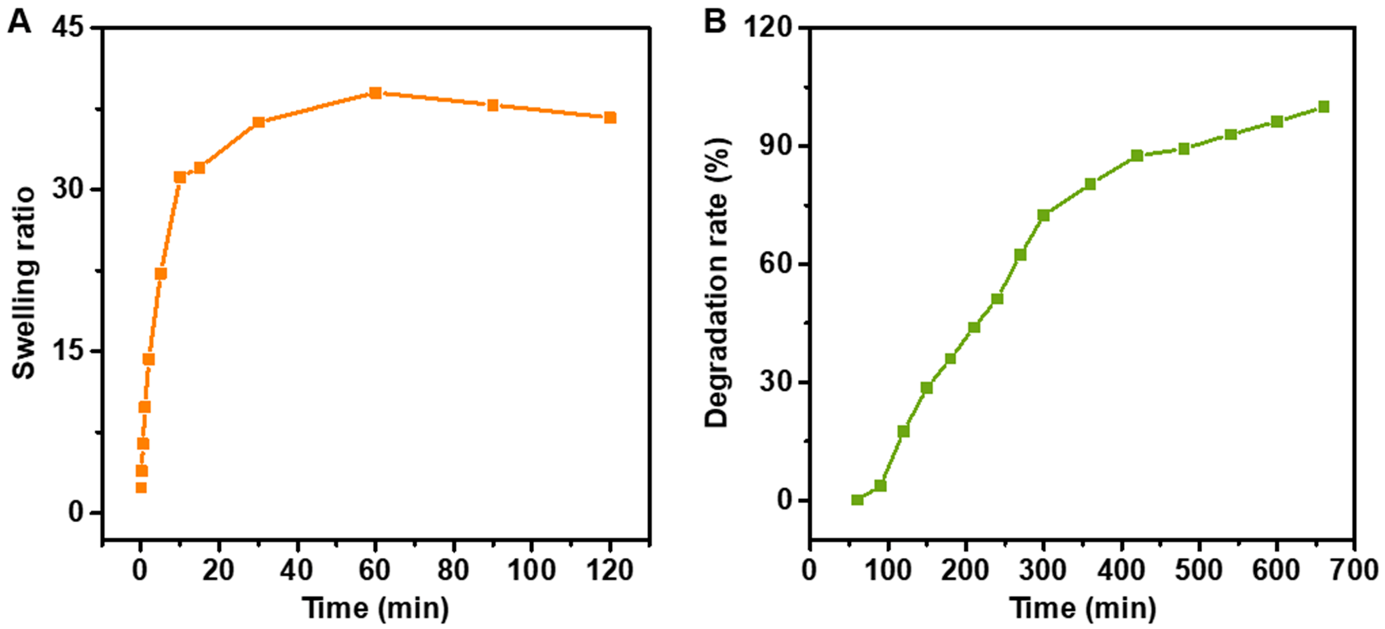
**

Figure S1. The swelling ratio of PDM (A) and degradation test of PDM in PBS solution (B).

Alt text: Data on the swelling ratio of PDM and degradation test of PDM in PBS solution, respectively.

**Figure S2**


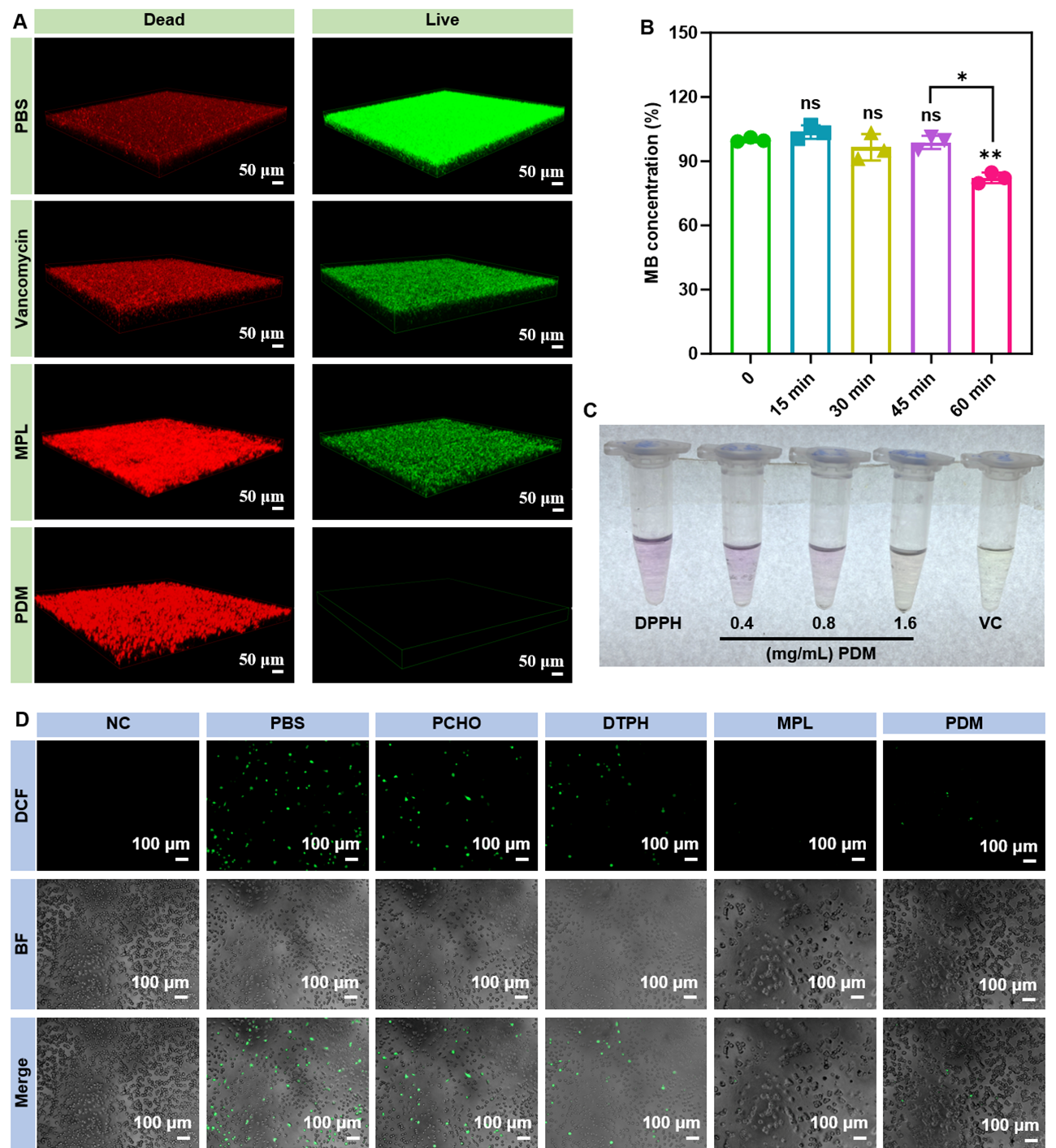


**Figure S2**. (A) The LIVE/DEAD staining of MRSA biofilm after different treatments (live bacteria: green; dead bacteria: red; scale bar: 50 µm). (B) Time-dependent degradation of MB triggered by Fenton reaction with PDM. (C) Immunofluorescence staining of ROS scavenging after treated by PDM and controls. ROS was stained as green (scale bar: 100 µm). Data were presented as mean ± SD. Statistical analysis of (B) performed using variance (ANOVA) followed by Tukey’s multiple comparison test (*p < 0.05, **p < 0.01 and ns means not significant differences. n = 3).

Alt text: Graphs and data on the LIVE/DEAD staining of MRSA biofilm, time-dependent degradation of MB, immunofluorescence staining of ROS scavenging in L929 cells.

**Figure S3**

**
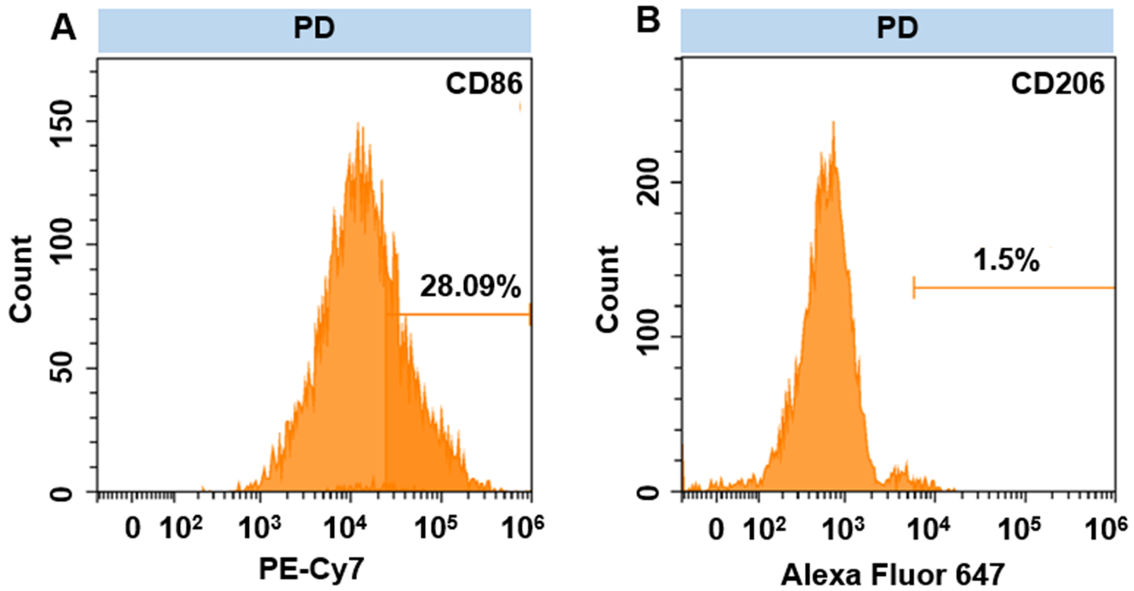
**

**Figure S3**. Effect of hydrogel matrix (PD) on M2 macrophages polarization. (A-B) Quantitative analysis of M1 macrophage (A) and M2 macrophages (B) after treated with PD by flow cytometry.

Alt text: Graphs on the Quantitative analysis of M1 macrophage (A) and M2 macrophages (B) after treated with PD by flow cytometry.

**Figure S4**

**
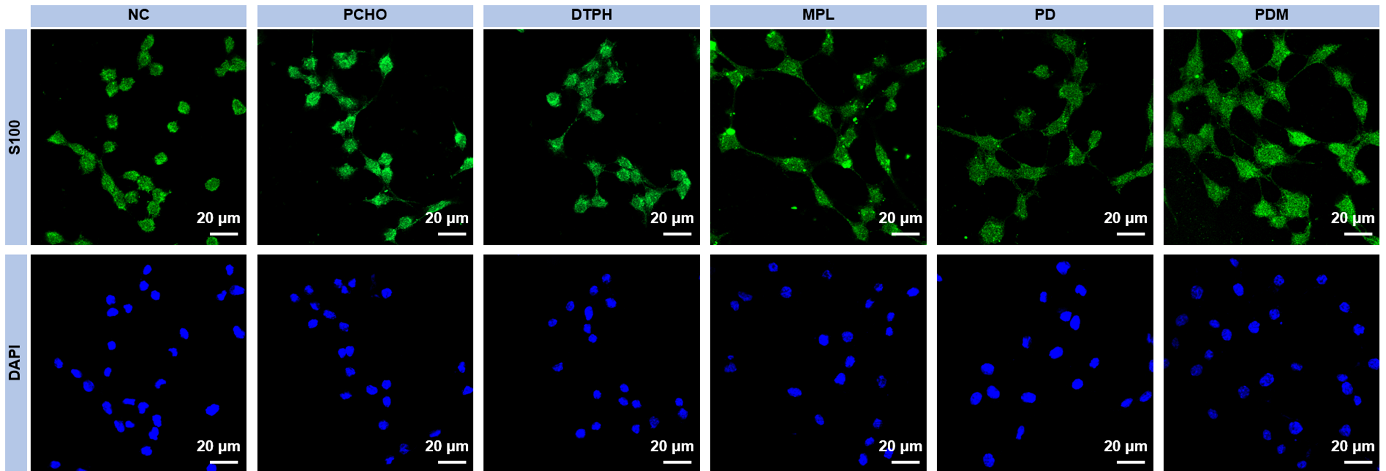
**

**Figure S4**. (A) Immunofluorescence staining of S100 in RSC-96 cells after treated with PDM solution or controls for 3 days, the NC group without any treatment was used as a control, S100 and nuclei were stained as green and blue, respectively (scale bar: 100 μm).

Alt text: Graphs on the immunofluorescence staining of S100.

**Figure S5**

**
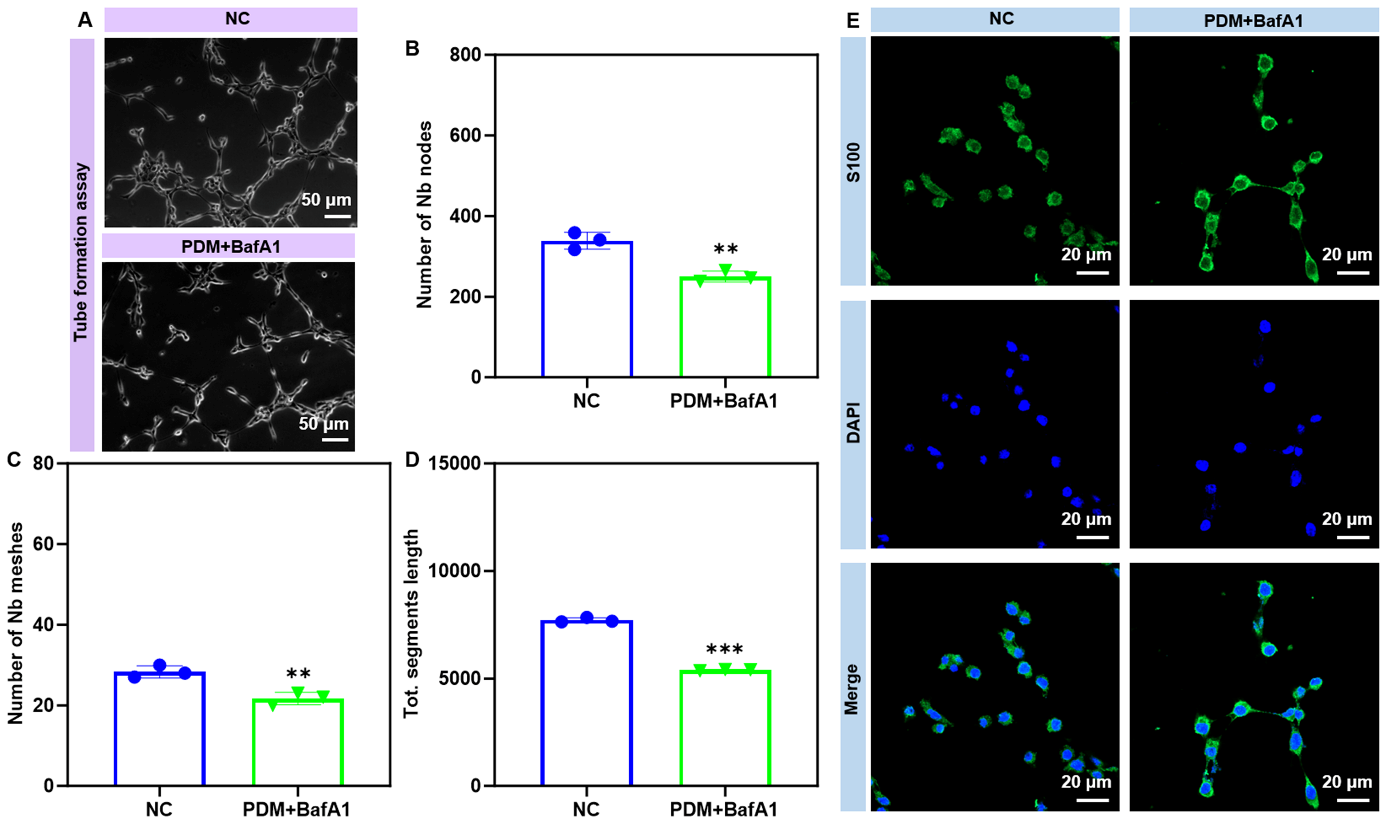
**

**Figure S5**. Influence of autophagy on the function of PDM in angiogenesis and Schwann cell maturation. (A) Images of HUVECs tubule formation after PDM solution with Bafilomycin A1 (BafA1) solution treatment for 8 h, Scale bars: 50 μm. (B-D) Quantitative analysis of number of Nb nodes (B), number of Nb meshes (C) and total segments length (D). (E) Immunofluorescence staining of S100 in RSC-96 cells after treated with PDM and BafA1 solution, S100 and nuclei were stained as green and blue, respectively (scale bar: 20 μm). Cells without any treatment were used as negative control (NC).

Alt text: Graphs and data on *in vitro* angiogenesis and Schwann cell maturation of PDM under autophagy inhibition, with subfigures labelled from A to D illustrating images and quantitative analysis of HUVECs tubule formation, E immunofluorescence images of RSC-96 cells after treated with PDM and Bafilomycin A1 (BafA1).

**Figure S6**

**
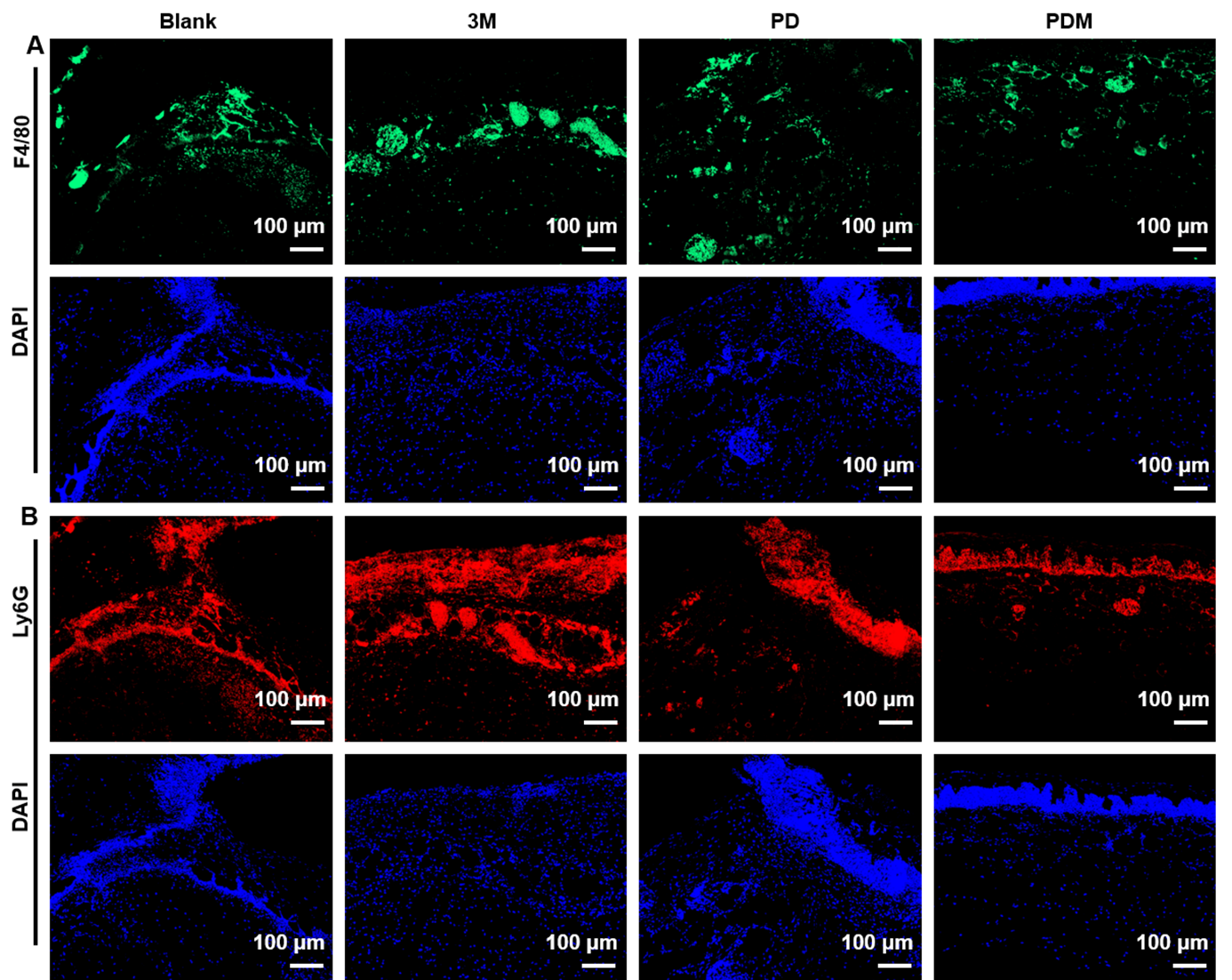
**

**Figure S6**. Immunofluorescence staining images of different groups on day 3. (A-B) F4/80 (green) and Ly6G (red) staining images, respectively, nuclei were stained as blue.

Alt text: Graphs on the immunofluorescence staining of F4/80 and Ly6G.

**Figure S7**


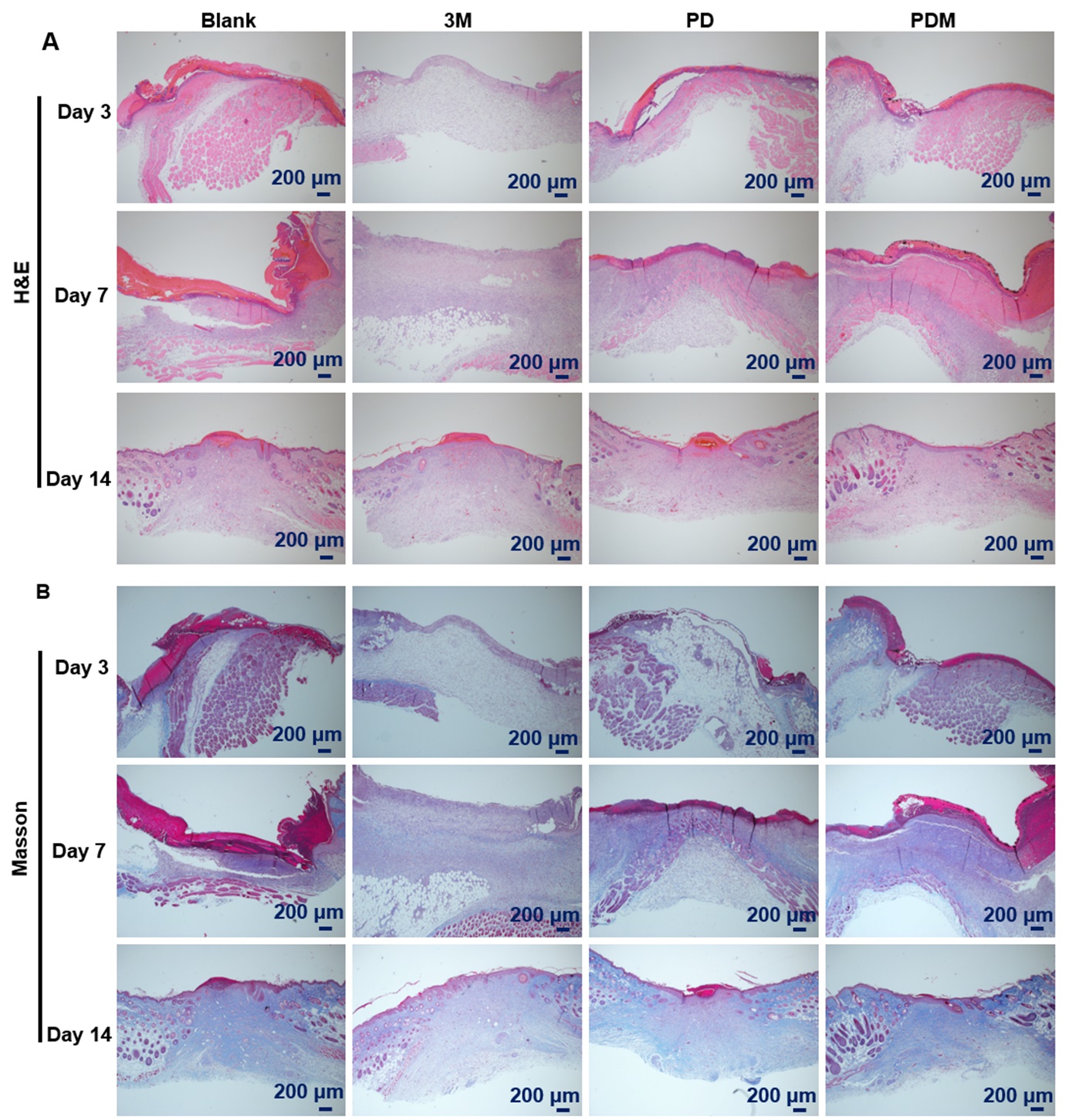


**Figure S7.** H&E and Masson’s trichrome staining of wounds after treated with PDM hydrogel and controls for 3, 7 and 14 days. Scale bar is 200 µm.

Alt text: Graphs on H&E and Masson’s trichrome staining.

**Figure S8**


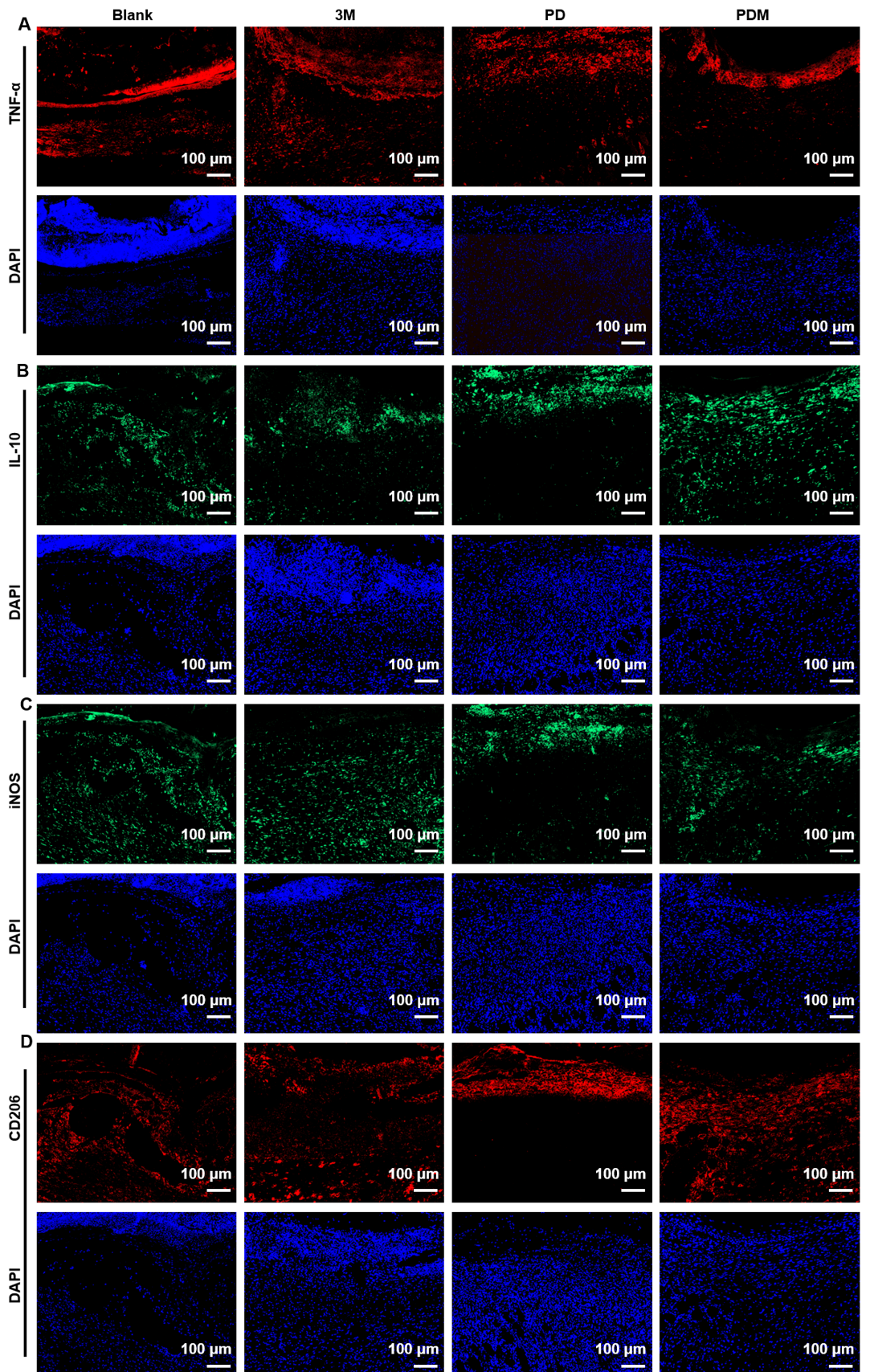


**Figure S8**. Immunofluorescence staining images of different groups on day 7. (A-D) TNF-α (red), IL-10 (green), iNOS (green) and CD206 (red) staining images, respectively, nuclei were stained as blue.

Alt text: Graphs on the immunofluorescence staining of TNF-α, IL-10, iNOS and CD206.

**Figure S9**


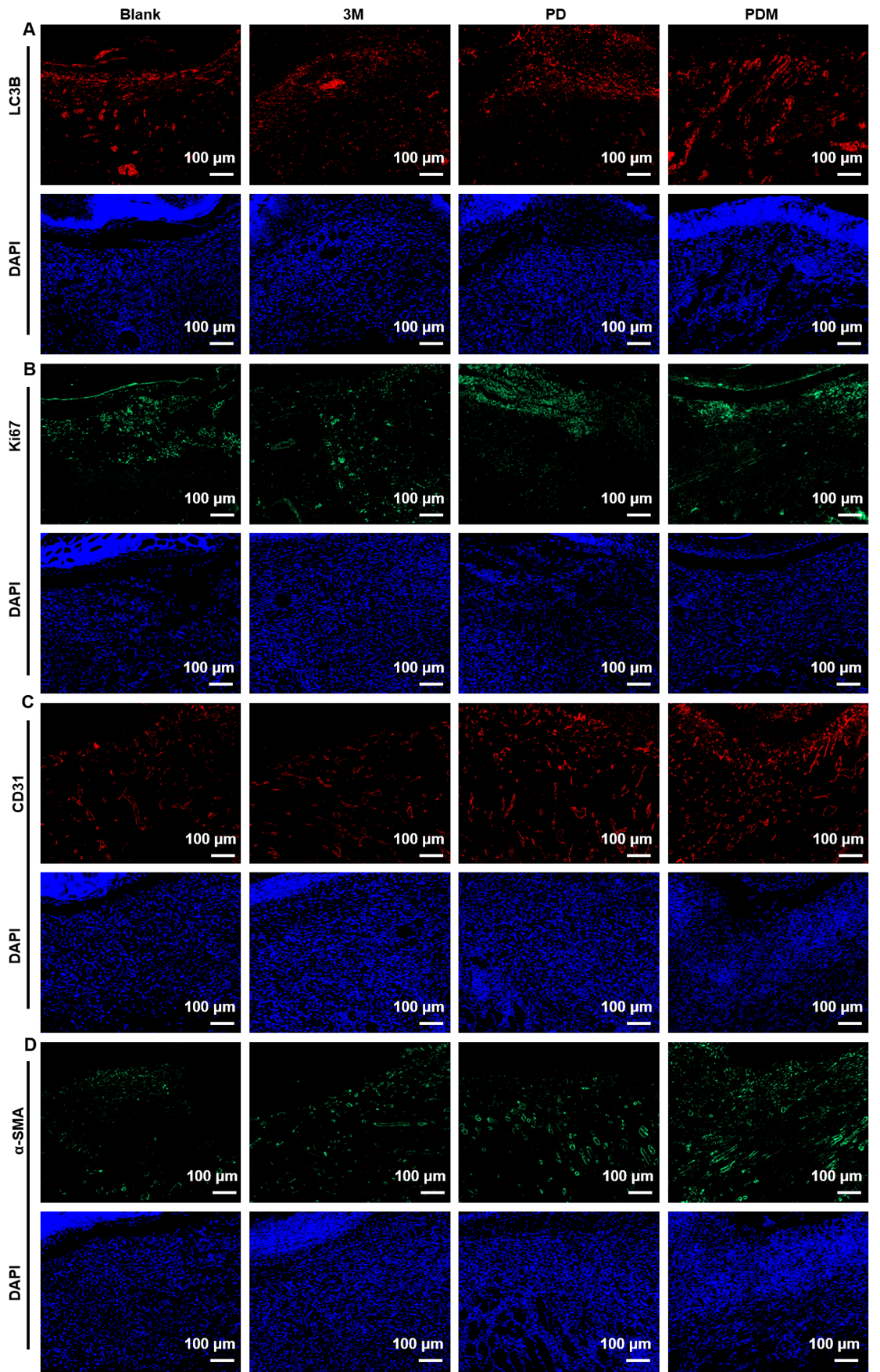


**Figure S9**. Immunofluorescence staining images of different groups on day 7. (A-D) LC3B (red), Ki67 (green), CD31 (red) and α-SMA (green) staining images, respectively, nuclei were stained as blue.

Alt text: Graphs on the immunofluorescence staining of LC3B, Ki67, CD31 and α-SMA.

**Figure S10**


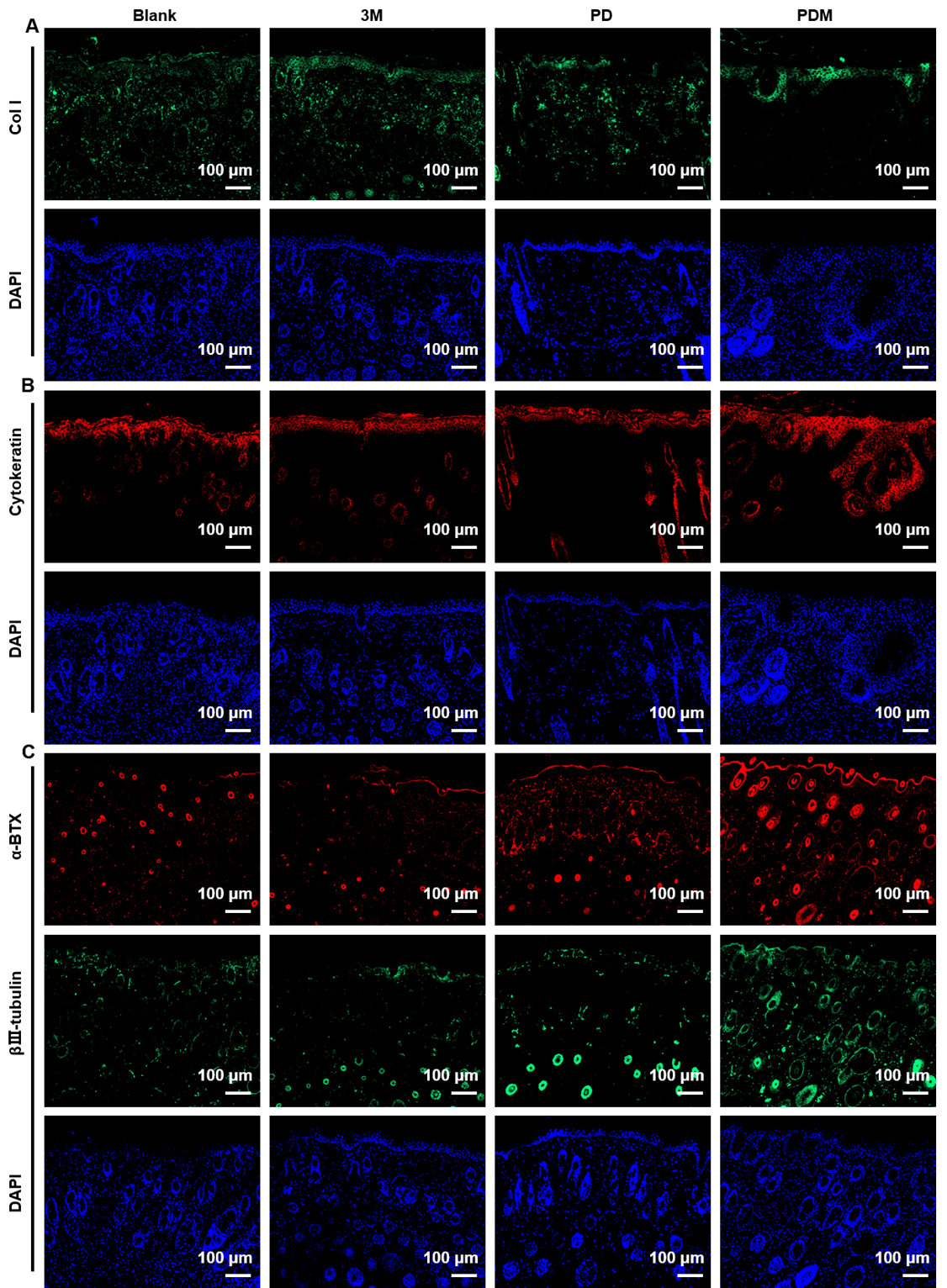


**Figure S10**. Immunofluorescence staining images of different groups on day 14. (A-B) Col Ⅰ (green) and Cytokeratin (red) staining images, respectively. (C) α-BTX (red) and βIII-tubulin (green) staining images, nuclei were stained as blue.

Alt text: Graphs on the immunofluorescence staining of Col Ⅰ, Cytokeratin, α-BTX and βIII-tubulin.

**Reference**

1. Sun C, Wang D, Zhang M, Ni Y, Shen X, Song Y, Geng Z, Xu W, Liu F, Mao C. Novel L-lactic acid biosensors based on conducting polypyrrole-block copolymer nanoparticles. Analyst 2015;140:797-802.

2. Yang J, Zhang X, Liu H, Wang C, Liu S, Sun P, Wang L, Liu Y. Heterostructured TiO_2_/WO_3_porous microspheres: Preparation, characterization and photocatalytic properties. Catal Today 2013;201:195-202.

3. Yang Z, Zheng H, Yin H, Zhou L, Zhang Q, Zhang B. Niobium carbide doped ROS/temperature dual-responsive multifunctional hydrogel for facilitating MRSA-infected wound healing. Chem Eng J 2023;471.

4. Zhou L, Zheng H, Liu Z, Wang S, Liu Z, Chen F, Zhang H, Kong J, Zhou F, Zhang Q. Conductive antibacterial hemostatic multifunctional scaffolds based on Ti_3_C_2_T_x_ MXene nanosheets for promoting multidrug-resistant bacteria-infected wound healing. ACS Nano 2021;15:2468-2480.

5. Hur J, Im K, Kim SW, Kim J, Chung D-Y, Kim T-H, Jo KH, Hahn JH, Bao Z, Hwang S, Park N. Polypyrrole/agarose-based electronically conductive and reversibly restorable hydrogel. ACS Nano 2014;8:10066-10076.
